# Optimization and Validation of CO_2_ Laser-Machining Parameters for Wood–Plastic Composites (WPCs)

**DOI:** 10.3390/polym17162216

**Published:** 2025-08-13

**Authors:** Sharizal Ahmad Sobri, Teoh Ping Chow, Tan Koon Tatt, Mohd Hisham Nordin, Andi Hermawan, Mohd Hazim Mohamad Amini, Mohd Natashah Norizan, Norshah Afizi Shuaib, Wan Omar Ali Saifuddin Wan Ismail

**Affiliations:** 1 Department of Engineering, Nottingham Trent University, Clifton Campus, Nottingham N11 8NS, UK; 2 School of Technology and Engineering Science, Wawasan Open University, 54 Jalan Sultan Ahmad Shah, George Town 10050, Pulau Pinang, Malaysia; pcteoh@wou.edu.my (T.P.C.); seantan@wou.edu.my (T.K.T.); 3 Faculty of Mechanical Technology and Engineering, Universiti Teknikal Malaysia Melaka, Hang Tuah Jaya, Durian Tunggal 76100, Melaka, Malaysia; hisham@utem.edu.my; 4 Laboratory of Wood Material Technology, Faculty of Agriculture, Kyushu University, 744 Motooka, Nishi-ku 819-0395, Fukuoka, Japan; andi.hermawan.151@m.kyushu-u.ac.jp; 5 Faculty of Bioengineering and Technology, Universiti Malaysia Kelantan, Jeli Campus, Jeli 17600, Kelantan, Malaysia; hazim.ma@umk.edu.my; 6 Tropical Wood and Biomass Research Group, Faculty of Bioengineering and Technology, Universiti Malaysia Kelantan, Jeli Campus, Jeli 17600, Kelantan, Malaysia; 7 Faculty of Electronic Engineering and Technology, Universiti Malaysia Perlis, Pauh Putra Campus, Arau 02600, Perlis, Malaysia; mohdnatashah@unimap.edu.my; 8 Centre of Excellence Geopolymer & Green Technology (CEGeoGTech), Universiti Malaysia Perlis, Kangar 01000, Perlis, Malaysia; norshahafizi@unimap.edu.my; 9 Faculty of Mechanical Engineering and Technology, Universiti Malaysia Perlis, Pauh Putra Campus, Arau 02600, Perlis, Malaysia; 10 Faculty of Islamic Contemporary Studies, Universiti Sultan Zainal Abidin, Gong Badak Campus, Kuala Nerus 21300, Terengganu, Malaysia; woasaifuddin@unisza.edu.my

**Keywords:** laser-cutting optimization, wood–plastic composites (WPCs), heat-affected zone (HAZ), statistical modelling, surface quality improvement

## Abstract

Wood–plastic composites (WPCs) offer a sustainable alternative to solid wood, yet their heterogeneous structure presents challenges in laser machining due to thermal sensitivity and inconsistent material behaviour. This study investigates the optimization of CO_2_ laser-cutting parameters for WPCs, focusing on feed rate and assist-gas pressure. Using a 1500 W CO_2_ laser, a full factorial experimental design was employed to cut 18 mm thick WPC panels at varying feed rates (1000–3000 mm/min) and gas pressures (1–3 bar). Statistical analyses including MANOVA and linear regression were conducted to evaluate their effects on key machining responses: cutting depth, heat-affected zone (HAZ) width, cut-edge quality, and surface finish. Results indicated that feed rate significantly influences both cutting depth and thermal damage, while gas pressure plays a major role in improving surface quality and reducing HAZ. Optimal combinations were identified for various performance goals, and validation trials at the selected parameters confirmed alignment with predicted outcomes. The optimized settings yielded high-quality cuts with reduced HAZ and enhanced surface characteristics. This study demonstrates the effectiveness of a statistical optimization approach in refining CO_2_ laser-cutting conditions for WPCs, offering insights for improved process control and sustainable manufacturing applications. This study also introduces a multi-objective optimization approach that verifies the interaction effects of feed rate and assist-gas pressure, enabling precise and efficient CO_2_ laser cutting of 18 mm thick WPCs.

## 1. Introduction

Wood–plastic composites (WPCs) have gained widespread use in construction and manufacturing due to their wood-like appearance, durability, and sustainability [1,2]. Composed of wood fibres embedded in a thermoplastic matrix such as recycled plastics, WPCs offer the strength of wood combined with the rot-proof and recyclable benefits of polymers [3]. However, their heterogeneous structure and thermal sensitivity present challenges for machining processes, particularly laser cutting [4]. If not carefully controlled, prolonged laser dwell time or excessive heat input can result in thermal damage in WPCs, such as charring, delamination, or discoloration of the wood fibres and polymer matrix [5,6]. Optimizing laser-cutting parameters for WPCs is therefore essential to achieving clean cuts with minimal heat-affected zones and material degradation [7,8].

CO_2_ laser cutting is a popular non-contact method that offers high precision and the ability to cut complex shapes in a variety of materials, including wood and polymer composites. During laser cutting, a focused high-power beam locally heats, melts, and vaporizes the material along the cut path. The quality of laser cuts is influenced by operational parameters such as cutting speed (feed rate), laser power, and assist-gas type/pressure, in addition to material properties like density, thickness, and thermal conductivity [9]. For wood-based materials and natural fibre composites, numerous studies have highlighted cutting speed and assist-gas pressure as key factors determining kerf width, surface charring, and HAZ extent. Generally, a higher cutting speed reduces the energy delivered per unit length, thereby narrowing the kerf and HAZ by limiting thermal exposure time. For example, Eltawahni et al. [10] noted that laser cutting of medium-density fibreboard (MDF) is an exothermic process (i.e., the burning of wood components can exacerbate thermal damage) making it imperative to minimize the laser residence time to reduce charring and HAZ. In various thermoplastic composites, cutting speed has indeed been identified as one of the most influential factors on HAZ size, with higher speeds significantly decreasing the depth and width of the HAZ by limiting heat build-up [11,12]. Conversely, excessive laser power or very slow feed rates often lead to extensive charring and a wider HAZ, as prolonged heat input degrades the material [12,13]. Overall, past research indicates that using the minimum laser energy and maximum travel speed necessary to achieve the cut can minimize thermal damage in the laser machining of wood and composites [11,14].

Assist-gas pressure is another critical parameter in laser cutting, especially for organic or polymer-based materials. The assist gas (often compressed air, nitrogen, or other inert gas) is directed coaxially at the cutting zone to blow away molten material and combustion byproducts from the kerf, while also providing cooling to the cut front. Adequate gas pressure can prevent excessive burning by displacing oxygen and quenching combustion at the material surface. For example, Liu et al. [15] found that adding a high-flow inert gas (helium) during CO_2_ laser cutting of thin wood drastically reduced edge burning and produced cleaner kerfs by isolating the cut zone from oxygen. Similarly, studies on natural fibre-reinforced polymers have shown that increasing assist-gas pressure narrows the HAZ and improves cut surface quality by reducing oxidation and cooling the material [12,13]. In one study, Masoud et al. [12] reported that, for a 2–6 mm thick natural fibre polyester composite, gas pressure was the most significant factor influencing HAZ dimensions, whereas traverse speed became increasingly important at greater thicknesses, and laser power had a comparatively minimal effect on HAZ. These findings underscore the important role of assist gas in achieving clean cuts, especially in combustible composite materials.

Machining and processing natural fibre composites, such as WPCs, require specialized attention due to their heterogeneous structure and the presence of natural fibre reinforcements. Hybrid natural fibre composites, for instance, exhibit machining challenges due to variations in fibre distribution, density, and thermal conductivity, which influence cut quality and mechanical integrity [16]. Effective machining strategies must therefore be adapted specifically to the unique characteristics of the composite materials to minimize damage, maximize surface finish, and sustain structural properties.

Research on the formulation and preparation of sustainable WPCs underscores their environmental potential, utilizing fibres extracted from agricultural and municipal solid waste, such as durian husk fibre and recycled polystyrene foam [17,18]. These WPCs not only enhance waste recycling practices but also present viable substitutes for conventional solid wood and plastics. Despite these sustainability benefits, inherent variability in fibre properties poses challenges for consistent machining processes, necessitating detailed investigation into the influence of machining parameters on final composite properties.

Emerging techniques, such as additive manufacturing (AM), have opened new avenues for processing natural fibre reinforced polymer composites [19]. However, conventional subtractive machining techniques like laser cutting remain prevalent in industrial applications requiring precision and cost efficiency, particularly when handling thick composite panels. Investigations into drilling WPCs, for instance, have demonstrated that machining parameters critically influence defects such as delamination, emphasizing the importance of selecting optimal settings to minimize structural damage and enhance overall material performance [20].

Despite these insights, the combined interplay between feed rate and assist-gas pressure in CO_2_ laser cutting of WPCs has not been fully explored in prior literature. Many published studies focus on single-factor effects or use relatively thin composites. A comprehensive approach that examines not only the individual effects of feed rate and gas pressure but also their interaction on multiple quality responses (such as cut depth, HAZ width, and surface finish) is lacking for thick WPC materials. Addressing this gap is important because WPCs present a multi-faceted machining challenge: one must balance the need for sufficient laser energy to cut through the material (18 mm thick in this case) against the need to minimize thermal damage to the cut edges. Optimizing these laser parameters can broaden the applicability of laser cutting for sustainable composite materials by improving cut quality and reducing post-processing needs.

While previous studies have investigated laser cutting of medium-density fibreboard (MDF) [10] and natural fibre-reinforced polymer composites [12,13], these works generally focus on relatively thin sections (≤6 mm) and often examine single-factor effects without validating parameter interactions. For instance, Eltawahni et al. [10] optimized CO_2_ laser-cutting parameters for MDF and reported that cutting speed strongly influenced HAZ formation, but their study did not address the combined influence of assist-gas pressure and feed rate, nor did it extend to thick composite sections. Similarly, Masoud et al. [12] analyzed gas pressure effects on natural fibre polyester composites and found it to be the most significant factor in reducing HAZ, yet their work was limited to thin laminates and lacked a multi-objective optimization framework. In contrast, the present study uniquely investigates 18 mm-thick WPC panels, quantifies the interaction effects between feed rate and assist-gas pressure across multiple quality metrics, and applies a multi-objective optimization and validation strategy. This approach provides the first comprehensive, experimentally validated parameter matrix for high-precision CO_2_ laser machining of thick WPCs, bridging a gap in the literature and offering actionable process guidelines for industrial applications.

Liu et al., (2020) [15] investigated CO_2_ laser cutting of thin wood under a high-flow *inert* assist-gas environment (helium) and showed that isolating the cut front from oxygen markedly suppressed edge burning and produced cleaner, narrower kerfs. Their experiments emphasized the role of assist-gas composition and flow in limiting oxidative charring, demonstrating that gas delivery can dominate visual quality even when beam parameters are unchanged. However, the study was confined to thin wood sections and did not consider thicker hybrid materials or quantify feed-rate × gas interactions across multiple responses (e.g., HAZ, surface finish, penetration).

This paper aims to optimize and validate the CO_2_ laser-cutting parameters for thick WPCs through a rigorous experimental and statistical approach. Building on initial experimental findings that identified feed rate and assist-gas pressure as key factors, predictive models are developed for multiple cut quality responses and use these models to determine optimal parameter combinations for different cutting objectives. The ultimate goal is to establish data-driven guidelines for laser cutting of WPCs that maximize cutting efficiency (depth of cut) while minimizing thermal damage (HAZ and surface char), thereby improving the feasibility and quality of laser processing for these sustainable composite materials. To ensure practical relevance, the optimized settings are validated by confirmatory trials, providing empirical evidence that the chosen parameters indeed yield improved performance. Building on these insights, the present work targets thick (18 mm) wood–plastic composites (WPCs) and explicitly quantifies the *interaction* between feed rate and assist-gas pressure across multiple responses (cut depth, HAZ, edge profile, surface finish). In contrast to prior thin-section studies, this article (i) map a 3 × 3 factorial space at constant 1500 W, (ii) apply MANOVA plus per-response ANOVA and linear models to capture trade-offs, and (iii) perform validation trials at the recommended conditions to confirm predictive accuracy. The resulting parameter matrix provides actionable guidance for high-precision CO_2_ laser machining of thick WPCs and shows when depth (slow feed) must be traded for quality (fast feed + high gas).

## 2. Materials and Methods

### 2.1. Materials and Equipment

The work material for this study was a wood–plastic composite (WPC) panel of 18 mm thickness, which it was sourced from local supplier in Kuala Lumpur, Malaysia. The WPC consisted of approximately 60% wood fibre and 40% thermoplastic (recycled high-density polyethylene, HDPE) by weight. The panels were manufactured for construction-grade use, resulting in a dense composite with wood-like mechanical properties and plastic-like moisture resistance. All test samples were cut from a single WPC panel to ensure batch consistency and minimize material variability. To ensure batch consistency and minimize material variability, all specimens were taken from the same production batch. Material homogeneity (no outliers were observed and the batch was deemed uniform for machining trials) was verified by assessing the density distribution across five random cross-sections using the water displacement method in accordance with ASTM D792, i.e., density was measured in-house by water-displacement per standard on five randomly selected cross-sections cut from the same production batch. Table 1 summarizes the key characteristics of the WPC material, including its composition, density, and other relevant specifications.

**Figure 1:**
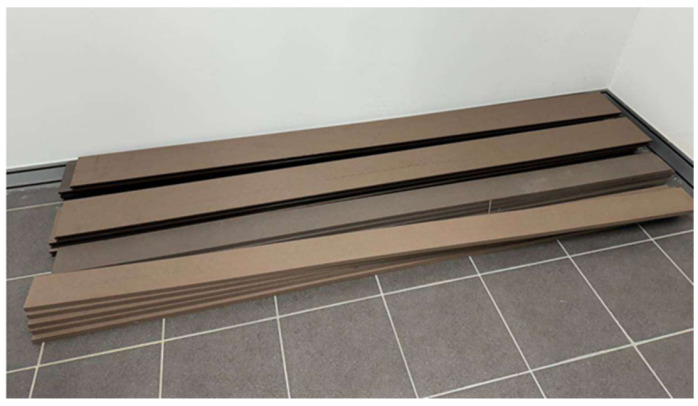
Wood–plastic composites (WPCs) before converting into smaller samples.

Prior to laser cutting, the WPC panels were cut into smaller triangular samples measuring 30 mm × 40 mm with a thickness of 18 mm to facilitate handling and examination. Figure 1 shows the work material. A triangular specimen geometry was purposefully selected to capture variations in thermal and mechanical responses across different directional edges. The triangular shape introduces distinct thermal gradients and stress concentrations, particularly at corner regions, making it an effective test case for evaluating consistency in laser-cutting performance, kerf morphology, and heat-affected zone development. Previous studies have shown that specimen geometry (including triangular profiles) can significantly affect thermal stress distribution and material responses during laser cutting. For example, Yilbas et al. [21] investigated laser cutting of triangular geometries in aluminum alloy and found pronounced von Mises stress concentrations near the triangle corners, influenced by size and shape, which directly affected cut quality and edge morphology. By incorporating triangular WPC samples, this study enriches the model validation and ensures that the optimization strategy remains applicable for components with irregular geometry commonly encountered in real-world applications.

All laser-cutting experiments were conducted using a CO_2_ laser-cutting system equipped with an industrial-grade laser rated at 1500 W and operating at a wavelength of 10.6 µm. The laser functioned in continuous-wave (CW) mode with a Gaussian beam profile. A focusing lens with a focal length of 50.8 mm produced a focused spot approximately 0.3 mm in diameter on the surface of the workpiece. The laser head was fitted with an assist-gas nozzle (2.3 mm inner diameter), which directed the gas coaxially with the beam onto the cutting zone. Compressed air (i.e., closely approximating the atmospheric mix of nitrogen and oxygen) was used as the assist gas, delivered at controlled pressure levels.

WPC samples were positioned on a computer-controlled XY motion stage, enabling automated cutting at predefined feed rates. The laser beam was aligned perpendicular to the surface of the samples (normal incidence), and the focal point was set at the mid-thickness of the panel (approximately 9 mm from the top surface) to optimize cut quality at both the entry and exit points. The laser was operated at a constant power setting of 1500 W in CW mode throughout the experiments. This power level was selected to ensure the capability for full penetration through the 18 mm thick WPC panels, particularly at lower feed rates.

**Figure 2:**
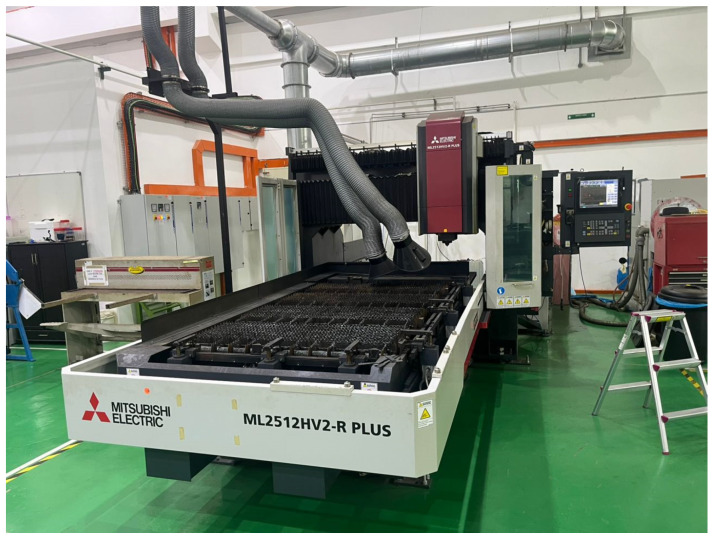
Mitsubishi model ML32XP Laser CO_2_ Machining Centre.

The laser-machining experiments were carried out using the Mitsubishi CO_2_ 2D Laser Processing System, model ML32XP from the HV2-R Series, located at the Faculty of Mechanical Engineering Technology, Universiti Teknikal Malaysia Melaka (UTeM), Durian Tunggal, Malaysia. This industrial-grade system is equipped with a 3-axis SD excitation cross gas flow resonator, offering a rated power of 3.2 kW, beam mode TEM01, and power stability of ±1%. Integrated with high-pressure nitrogen and air assist-gas controls, the ML32XP enables high-precision processing performance. Figure 2 shows the laser-cutting equipment employed in this study. Additionally, the system is supported by an LCU15AIX cooling unit and a beam delivery mechanism featuring an Auto Focus preset head (PH-XS), facilitating accurate kerf formation and consistent depth penetration.

The selection of 1500 W was based on preliminary penetration trials and reference to prior studies on laser machining of wood-based composites of comparable thickness. Earlier work on MDF and natural fibre composites (e.g., Eltawahni et al. [10], Masoud et al. [12]) showed that full penetration of panels thicker than 12 mm typically required a minimum of 1200 W CO_2_ laser power under continuous-wave operation. In our preliminary tests on 18 mm WPC panels, power levels below 1400 W were unable to achieve consistent full-depth cutting even at the slowest practical feed rates, often resulting in incomplete kerf formation and excessive heat-affected zones due to prolonged dwell times. Therefore, 1500 W was selected as an operational balance, i.e., high enough to enable single-pass penetration at lower feed rates while still within the safe operating range of the Mitsubishi ML32XP system. This choice also ensured experimental consistency by avoiding variations in beam mode quality or focal spot size that can occur at lower power fractions in industrial laser systems. In addition to that, the 1500 W setting allowed exploration of a wide feed rate range (1000–3000 mm/min) without exceeding thermal damage thresholds observed in preliminary runs, thus providing a controlled and reproducible parameter space for optimization.

**Figure 3:**
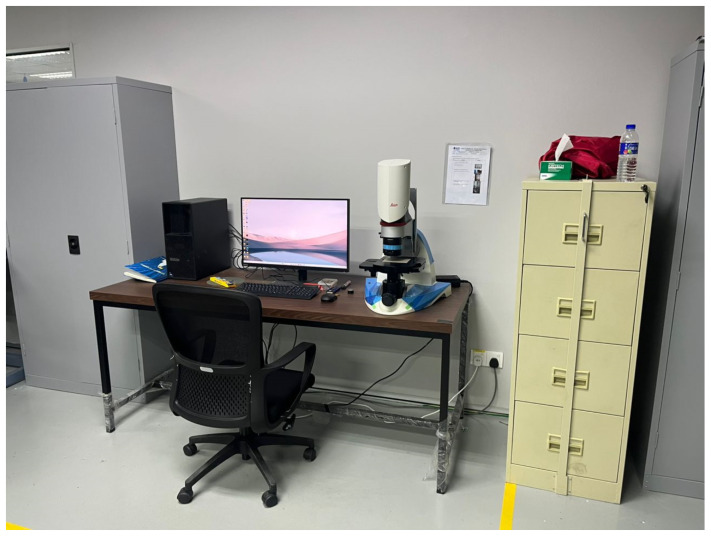
Leica DVM6 system digital microscope.

A Leica DVM6 digital microscope (Leica Microsystems, Wetzlar, Germany) was utilized for post-process quality assessment. This high-resolution imaging system features an integrated zoom module with PlanAPO objectives (FOV 12.55), providing magnification capabilities ranging from 10.04 mm × 7.53 mm down to 0.63 mm × 0.47 mm. The microscope is equipped with coaxial LED illumination and controlled via LAS X software, which enables precise image acquisition and dimensional measurements. A manual XY stage further supports detailed navigation across the sample surface. Figure 3 shows the Leica DVM6 system used for inspecting the surface characteristics and microstructural features of the WPC specimens.

Environmental and safety conditions were kept constant for all trials. Each WPC sample was clamped securely to avoid movement or warping during cutting, and ventilation was provided to exhaust the smoke and fumes generated by the laser-material interaction. All experiments were conducted in ambient room temperature (~25 °C) and low humidity conditions.

For each parameter combination, three independent replicates were performed. These replicates were taken from different WPC panels sourced from the same production batch to avoid bias from intra-panel variability. The panels were obtained from a single supplier and manufactured under controlled extrusion conditions to maintain consistency in density, moisture content, and wood-to-HDPE ratio (as reported in Table 1). Before laser cutting, all panels were conditioned at 20 ± 2 °C and 65 ± 5% relative humidity for at least 48 h to ensure moisture equilibrium. Panel homogeneity was further verified by measuring density at five random points on each panel using a precision balance and volume displacement method; variations were within ±1.5% of the batch mean, indicating uniform material properties across all samples.

### 2.2. Experimental Design

A full-factorial design of experiments was employed to investigate the effects of feed rate and assist-gas pressure on the laser-cutting performance. Two factors were varied: feed rate (also referred to as cutting speed) and gas pressure. In Table 2, each factor was tested at three levels representative of practical machining extremes and mid-range values.

These levels were chosen based on preliminary trials that indicated 1000 mm/min was the minimum speed required to achieve full depth on 18 mm WPC at 1500 W, and 3000 mm/min was near the upper limit of speed for cutting with acceptable edge quality at that power. Gas pressures above 3 bar did not show additional benefit in initial tests, and 1 bar was the lowest pressure at which the assist gas could be delivered reliably in the system.

The design thus comprised a 3 × 3 factorial matrix, with a total of 9 unique combinations of feed rate and gas pressure. To improve the statistical reliability of the results, each combination was replicated (multiple cuts were performed for each setting). In total, N = 27 cuts were made (each condition run three times) on identical WPC samples. The cut geometry for each trial was a straight line cut of 100 mm length across the sample, ensuring a consistent long cut for assessing surface quality and HAZ. Each cut was spaced sufficiently apart on the sample to avoid thermal overlap effects.

### 2.3. Response Measures and Data Collection

**Figure 4:**
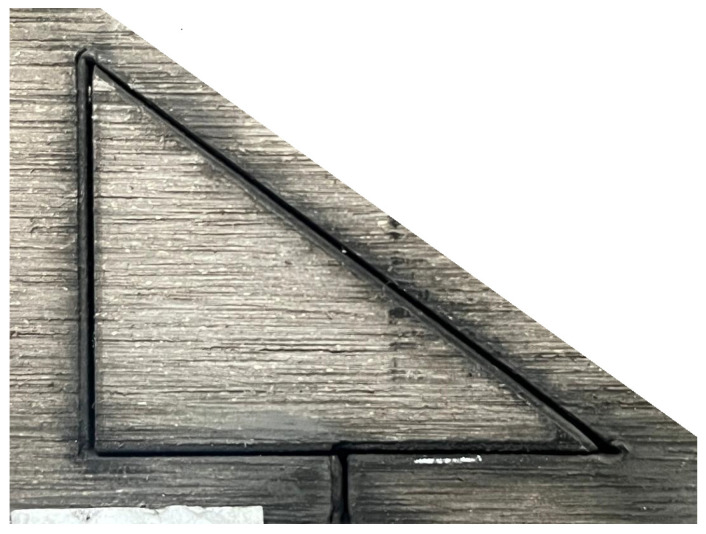
A cutting depth sample.

**Figure 5:**
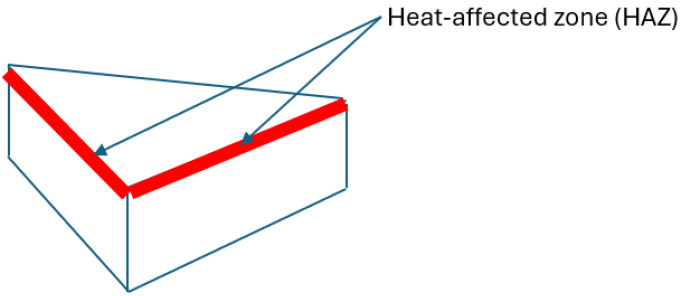
Visible HAZ region along the cut edge of the workpiece.

**Figure 6:**
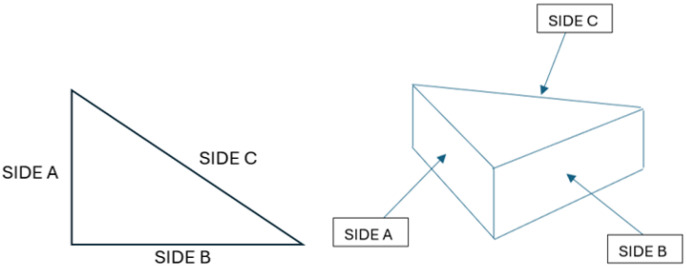
Evaluation of cut quality across three sides of the machined sample.

Four key response variables were measured for each cut, reflecting different aspects of cut performance and quality:

- *Cutting Depth:* The depth of the laser cut into the material, measured in millimetres (mm). If the laser fully cut through the 18 mm panel, the depth was recorded as 18 mm (full penetration). For cuts that did not fully penetrate, the depth of the kerf was measured by examining the cut cross-section. A thin feeler gauge (0.5 mm thickness) and a calliper were used initially to probe and measure depths, and for more precise measurement the samples were later cross-sectioned and examined under an optical microscope with a measurement reticle. The maximum depth of the kerf from the top surface was recorded to the nearest 0.5 mm. Figure 4 illustrates a representative WPC sample machined at a feed rate of 2000 mm/min, using a 1500 W CO_2_ laser and an assist-gas pressure of 1 bar.

- *Heat-Affected Zone (HAZ) Width:* The width of the thermally affected region adjacent to the cut, measured in millimetres. This was determined by visual inspection of the cut cross-sections and surface (see Figure 5), looking for the darkened or charred band along the cut edges. The HAZ width was measured on the top surface of the cut using a calibrated optical microscope (at 5× magnification) with an in-built scale. Two measurements were taken on each side of the kerf (if present) for each cut, and the average HAZ width on either side was recorded. In cases of full penetration, HAZ was measured at both the top and bottom cut edges. For consistency, the reported HAZ width refers to the top surface HAZ (which was typically larger than the bottom due to the focus position and gravity-assisted debris removal).

- *Cut-Edge Profile Quality:* A qualitative rating of the cut-edge geometry and integrity, focusing on kerf width and edge straightness/taper. After each cut, the cut face (profile) of the sample was inspected. Cuts that fully penetrated produced a detached piece, allowing direct observation of the cut edge; partial cuts were examined by cutting the sample to expose the kerf. The cut profile quality was scored on a subjective scale (1 to 5) based on smoothness and consistency of the kerf: a higher score indicated a straighter, smoother kerf with minimal taper or irregularities, whereas a lower score indicated irregular or tapered kerf profiles (e.g., top and bottom kerf misalignment or non-uniform width). Two independent observers scored each cut, and the average was taken as the cutting profile score.
- *Surface Finish Quality:* A qualitative assessment of the cut surface (the side walls of the kerf) in terms of roughness and charring. This was also rated on a 1 to 5 scale, where a higher score corresponds to a smoother, cleaner cut surface with little residue or char, and a lower score corresponds to a rough or heavily charred surface. Photomicrographs of the cut surfaces were taken using a digital microscope for documentation. Additionally, for cuts that did not go through, the bottom of the kerf was inspected to see if there was residual material or incomplete cutting. The surface quality rating emphasizes visual smoothness and absence of adhered debris. Figure 6 illustrates the evaluation of three sides.

It should be noted that the qualitative scores for cutting profile and surface finish serve as proxies for more quantitative measures (like kerf taper angle and surface roughness, respectively) given the limitations of measuring these aspects on the large, charred surfaces of WPC. These ratings were used consistently across all samples to allow statistical analysis. Cut depth was measured at three equidistant positions along each kerf per replicate using a ±0.01 mm digital calliper (9 measurements per condition: 3 points × 3 replicates). Reported values are mean ± standard deviation (SD) across those nine measurements; error bars in the depth plots correspond to ±1 SD. Where full penetration occurred (18 mm), SD = 0 by definition because all positions registered complete cut-through. For partial cuts, SDs remained small (≤0.15 mm), indicating good repeatability at a given feed rate and gas pressure.

### 2.4. Statistical Analysis and Optimization Procedure

The experimental data were analyzed using statistical techniques to determine the effects of feed rate and gas pressure and to develop predictive models for each response:

- *Multivariate Analysis of Variance (MANOVA):* A two-way MANOVA was first performed considering the two factors (feed rate and gas pressure) and the four response variables collectively. This multivariate approach tests whether there are overall differences in the vector of responses due to the factors. It is particularly appropriate here since the responses (depth, HAZ, profile, surface quality) are interrelated aspects of cut quality. The MANOVA results (Wilks’ Lambda, Pillai’s Trace, etc.) were used to confirm whether feed rate and gas pressure have a statistically significant impact on the combined outcomes, and whether there is a significant interaction between these factors.
- *Analysis of Variance (ANOVA):* Following a significant MANOVA, separate two-way ANOVAs were conducted for each response variable. This allowed identification of which factors significantly affect each individual metric. The ANOVAs included the main effects of feed rate and gas pressure and their interaction. Post hoc tests (Tukey’s HSD) were used to compare differences between specific levels (e.g., between 1000 vs. 2000 mm/min, or 1 bar vs. 3 bar) for responses where the factor effect was significant.
- *Regression Modelling:* To enable prediction and optimization, multiple linear regression models were developed for each response. The models are expressed in the form shown in Equation (1):

Response = β_0_ + β_1_ × (Feed rate) + β_2_ × (Gas pressure) + ε (1)

where β_0_ is the intercept, β_1_ and β_2_ are coefficients for feed rate (in mm/min) and gas pressure (in bar), respectively, and ε is the residual error. Feed rate and gas pressure were treated as continuous variables in these models. Although the true relationships may not be perfectly linear over a wide range, within the tested range the linear fit provided a good approximation (as evidenced by high R^2^ values). The regression coefficients indicate the direction and magnitude of influence of each factor on each response. For example, a negative β_1_ for HAZ would indicate that increasing feed rate (faster cutting) reduces HAZ width.

- *Optimization Strategy:* Using the regression models, an optimization analysis was performed to identify favourable combinations of feed rate and gas pressure for different objectives. Rather than a single “global” optimum, it was sought to determine parameter trade-offs that would optimize specific goals:
  1. *Maximum Cutting Depth*: Find conditions that maximize depth of cut (important for through-cutting thick WPC).
  2. *Minimum HAZ/Best Quality*: Find conditions that minimize HAZ and maximize surface/profile quality (important for achieving clean, precise cuts).
  3. *Balanced Performance*: Identify a middle-ground setting that provides a reasonable compromise between depth and cut quality, for cases where both penetration and quality are needed. These optimization targets were informed by the regression trends. Because the objectives conflict (e.g., deeper cuts require slower speed, but slower speed worsens HAZ), the optimal parameters differ depending on the priority. Therefore, a multi-objective optimization approach was implicitly taken by examining the regression surfaces and selecting points that best satisfy each objective. Formal multi-objective algorithms were not applied; instead, engineering judgement and model predictions were used to pinpoint recommended settings (which conveniently corresponded to or near the tested factor levels).
- *Validation Trials:* After determining the recommended parameter settings from the model (for each objective scenario), additional laser-cutting trials were conducted at those settings to validate the model predictions. These validation trials involved cutting new WPC samples using the optimal feed rate and gas pressure combinations, then measuring the same responses (depth, HAZ, etc.) to see if the outcomes match the predicted improvements. By comparing the validation results with model-predicted values and with the original experimental data, it can confirm whether the optimization truly yields better performance and ensure the results are reproducible.

All statistical analyses, including Multivariate Analysis of Variance (MANOVA), regression modelling, and post hoc tests, were conducted using IBM SPSS Statistics (version 29). Data visualization (e.g., interaction plots, residuals analysis, and prediction models) was performed using both SPSS and Microsoft Excel 365, while graphical formatting was finalized with OriginPro 2023 for clarity and publication quality. A significance level of α = 0.05 was used for hypothesis tests. The regression models were evaluated for goodness-of-fit using the coefficient of determination (R^2^) and analysis of residuals to ensure no major violations of linear model assumptions within the experimental range.

To check that high in-sample R^2^ values were not artefacts of overfitting, standard regression diagnostics and out-of-sample validation were performed. Diagnostics included residuals-versus-fitted plots, normal Q–Q plots, Shapiro–Wilk tests of residual normality, Breusch–Pagan tests for homoscedasticity, and influence analysis via Cook’s distance. Because the design contains 9 unique factor combinations with 3 replicates each, it was evaluated generalization using leave-one-combination-out cross-validation (LOCO-CV): in each fold, all replicates from one feed × gas cell were held out, models were fit to the remaining cells, and predictions were generated for the hold-out cell. Nine-fold CV metrics were also reported. Finally, to quantify parameter uncertainty with small-sample noise, we computed bootstrap 95% confidence intervals for regression coefficients by resampling at the cell level (1000 iterations). Full diagnostics (plots, CV metrics, and CIs) are provided in Supplementary Figure S1a–h and Tables S1 and S2. Furthermore, given the two-factor, nine-cell design and near-linear response behaviour within this window, a formal desirability/Pareto procedure would be methodologically equivalent to selecting along the fitted planes; therefore this research reports the three operating points that represent the practical corners of that trade-off.

## 3. Results

### 3.1. Experimental Results: Effects of Feed Rate and Gas Pressure

The experimental outcomes demonstrated clear trends in how feed rate and assist-gas pressure affect the laser cutting of WPCs. Table 3 summarizes the cutting depth achieved for each combination of feed rate and gas pressure. Each condition was run in triplicate. For every replicate, cutting depth was taken at three evenly spaced locations along the laser path using a ±0.01 mm digital calliper. The standard deviation computed from these nine readings (3 points × 3 replicates) captures both local (along-kerf) and between-replicate variability characteristic of WPC laser cutting. Reporting these statistics enables a clearer assessment of process reproducibility and consistency across feed-rate and assist-gas settings. Furthermore, each value is the average depth of cut; 18 mm indicates full penetration of the material (Gas pressure had no significant effect on depth). A slower feed rate dramatically increased the achievable depth of cut, while higher feed rates resulted in shallower cuts. In contrast, gas pressure had little to no effect on cutting depth in this experiment.

As seen in Table 3, at the lowest feed rate of 1000 mm/min the laser was able to fully cut through the 18 mm WPC panel (cutting depth ≈ 18 mm) under all tested gas pressures. In contrast, at the highest feed rate of 3000 mm/min, the cut penetrated only about half the thickness (approximately 9 mm depth) even with maximum gas pressure. The intermediate feed rate of 2000 mm/min produced partial penetration (~15 mm depth). These results confirm that feed rate is the dominant factor governing cutting depth: slower cutting speeds allow the laser more dwell time on a given area, delivering more energy and thus cutting deeper into the material, whereas faster speeds reduce the energy input per unit length, resulting in shallower cuts. Statistically, feed rate had a highly significant effect on depth (ANOVA *p* < 0.001), and post hoc comparisons indicated that all pairwise differences between the three feed levels were significant (e.g., 1000 vs. 2000 mm/min, 1000 vs. 3000 mm/min, etc., each yielding depth differences on the order of several millimetres). On the other hand, assist-gas pressure showed no significant main effect on cutting depth (*p* ≈ 0.99 for gas pressure in ANOVA), which is consistent with the observation that each feed rate yielded the same depth regardless of 1, 2, or 3 bar. The feed rate × gas pressure interaction effect was also not significant for depth, indicating that gas pressure did not influence depth even at different speeds. This is understandable because, unlike in some metal cutting scenarios, the assist gas in current WPC cutting primarily aids in cooling and debris removal rather than materially affecting the energy required to cut through the thickness.

**Figure 7:**
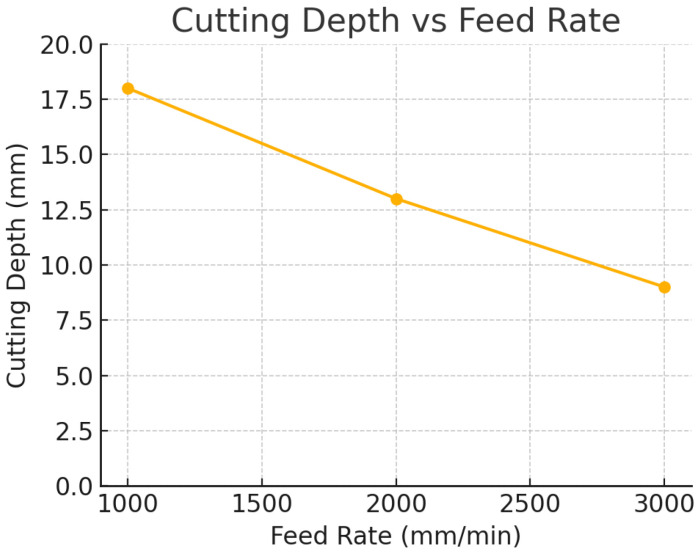
Plot of cutting depth vs. feed rate for a 1500 W CO_2_ laser cutting 18 mm WPC.

Feed rate and gas pressure both influenced the heat-affected zone (HAZ) width and cut quality metrics, though in different ways. Figure 7 illustrates the general relationship between feed rate and cutting depth, as discussed, but feed rate also had an inverse relationship with HAZ width. At the slowest feed (1000 mm/min), the HAZ around the cut was visibly larger, i.e., the edges of the cut were darkened over a wider band, indicating more extensive charring and thermal penetration into the material. At the fastest feed (3000 mm/min), the HAZ was much narrower; in fact, visual inspection and measurements indicated roughly a 50% reduction in HAZ width at 3000 mm/min compared to 1000 mm/min, all else equal. Quantitatively, the average top-surface HAZ measured ~1.0 mm wide per side at 1000 mm/min (with 1 bar gas), whereas at 3000 mm/min (with 1 bar) it was ~0.5 mm. The ANOVA results confirmed that feed rate had a significant effect on HAZ width (*p* < 0.001). Additionally, gas pressure exhibited a significant effect on HAZ (*p* < 0.001 for gas in ANOVA): higher gas pressures consistently led to narrower HAZ. For example, at 1000 mm/min, increasing the assist gas from 1 bar to 3 bar reduced the HAZ from about 1.0 mm to 0.7 mm. At 3000 mm/min, the HAZ was very small in all cases, but high gas still provided a slight reduction (e.g., from ~0.5 mm at 1 bar to ~0.3–0.4 mm at 3 bar). The cooling and flushing action of the gas likely prevented prolonged burning of the wood fibres, thus confining the thermal damage closer to the kerf.

**Figure 8:**
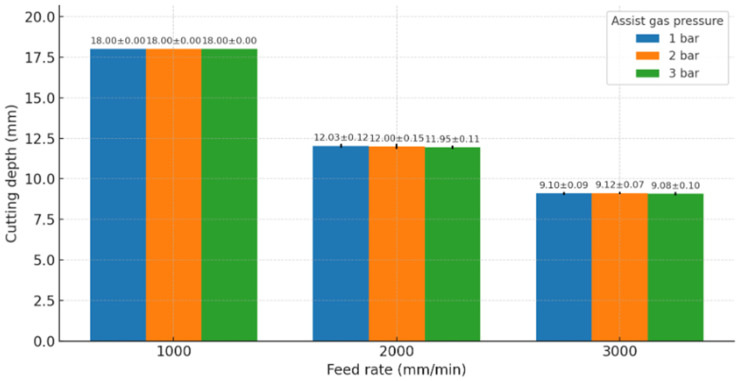
Cutting depth at 1500 W CO_2_ laser (mean ± SD; n = 3).

In Figure 7, assist-gas pressure 1–3 bar; gas pressure had negligible effect on depth and is not distinguished in this plot). Cutting depth decreases approximately linearly with increasing feed rate. Slower feed rates (1000 mm/min) achieved full penetration (18 mm), whereas faster feed rates (3000 mm/min) resulted in much shallower cuts (~9 mm) due to reduced energy input per unit length. Figure 8 re-plots cutting depth versus feed rate with error bars for each assist-gas level (1, 2, 3 bar). The overlap of the three pressure series at each feed rate and the very narrow error bars corroborate the ANOVA result that gas pressure had no measurable effect on depth within the tested range, whereas feed rate dominated penetration. Specifically, the variance at 2000 mm/min and 3000 mm/min is low (SD ≤ 0.15 mm), and at 1000 mm/min all three gas levels achieved full cut-through (18 mm, SD = 0). These outcomes are consistent with the regression model for depth (R^2^ = 0.964) and the previously reported non-significant gas main effect.

**Figure 9:**
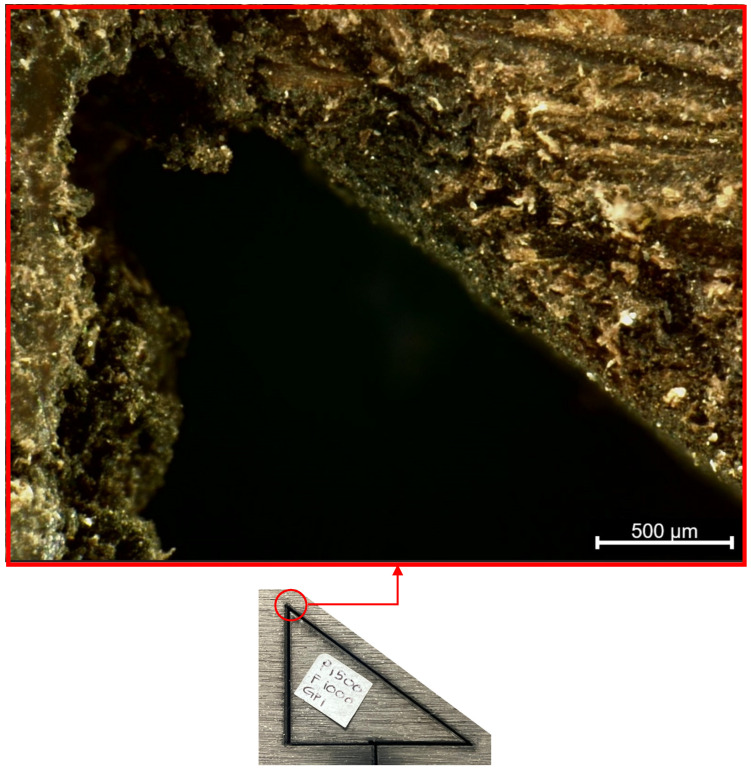
An up-close view of the cutting profile obtained at 1500 W laser power, with a feed rate of 1000 mm/min and 1 bar assist-gas pressure.

**Figure 10:**
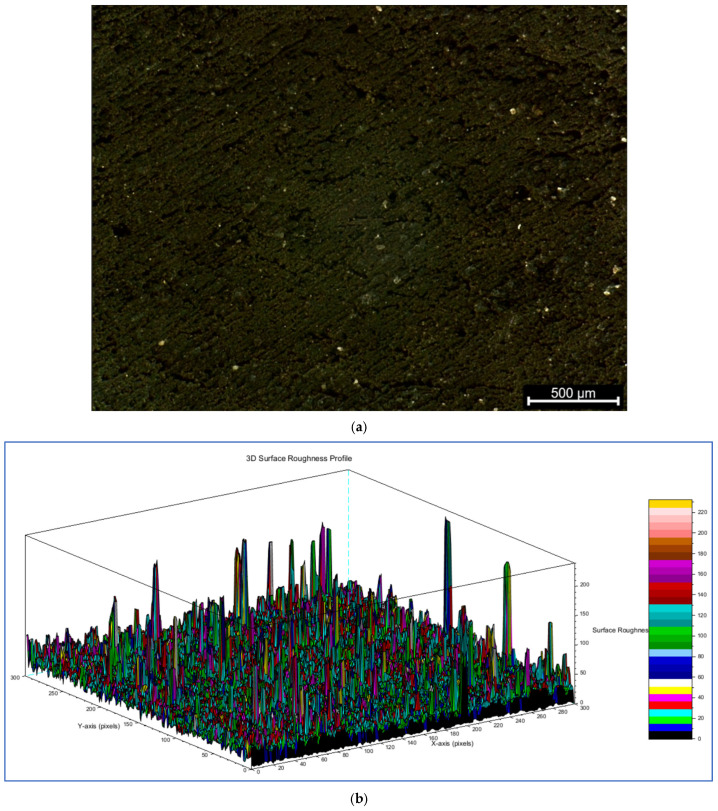
Surface profile of Side B at a feed rate of 1000 mm/min, 1500 W laser power, and 2 bar assist-gas pressure: (**a**) Cross-section view of surface profile; (**b**) three-dimensional surface roughness profile of Side B.

Feed rate and gas pressure also affected the cut-edge profile and surface finish of the cuts. Qualitatively, the edges produced at 1000 mm/min showed more pronounced thermal damage, i.e., they were often covered in a black charred layer with some adhered debris and resin. The cut kerf at 1000 mm/min tended to be wider at the top surface and slightly tapered (narrower) toward the bottom, likely due to excessive burning and melt collapse near the top where the laser lingered longest. In contrast, at 3000 mm/min, the cut edges were significantly cleaner and more uniform: minimal charring was observed, and the kerf width was more consistent from top to bottom (when penetration occurred) or in the portion that was cut. The surface of the kerf produced at high speed had a smoother appearance with striations indicative of the high traverse rate but little residue. At 2000 mm/min, the quality was intermediate, showing some char but less than at 1000 mm/min. Figure 9 presents a representative cut-edge profile, whereas Figure 10 displays a sample of the surface profile together with the 3D surface roughness profile.

These figures show the surface morphology of selected laser-cut edges of the wood–plastic composite (WPC) samples. Visual distinctions in char accumulation, edge melting, and surface texture are evident across varying process parameters. Cuts produced at lower feed rates (e.g., 1000 mm/min) demonstrate greater thermal damage, characterized by expanded heat-affected zones and darker edge coloration. In contrast, higher feed rates yield sharper, cleaner edges with less evidence of char deposition, indicating a reduced thermal load due to decreased laser exposure duration. The application of assist gas further contributed to edge clarity by mitigating the accumulation of molten debris. While quantitative surface roughness values (Ra or Rz) could not be obtained due to equipment limitations, a structured visual scoring method was applied to allow comparative analysis across different feed rate and gas pressure combinations. To enhance objectivity, comparative microscopic images were also utilized, offering higher resolution insight into surface topography across experimental trials.

Microscopic images of the cut kerf faces, as presented in Table 4, were captured at 500 µm scale from three distinct cross-sectional locations on the triangular WPC samples, all processed at a constant feed rate of 1000 mm/min. These views, i.e., designated as Sides A, B, and C, correspond to the laser entry point and lateral faces around the perimeter of the cut. The images are arranged by increasing assist-gas pressure (1 bar to 3 bar), enabling visual comparison of gas pressure effects under controlled cutting conditions. A consistent trend is evident: as gas pressure increases, the surfaces become visibly smoother and less contaminated. At 1 bar, surfaces show substantial darkening and residue buildup. At 2 bar, a noticeable reduction in char is observed, and at 3 bar, kerf surfaces appear significantly improved, exhibiting minimal soot and well-preserved wood-polymer textures.

The visual evidence supports the broader experimental findings and demonstrates that higher assist-gas pressures facilitate improved surface finish by effectively ejecting molten material and limiting oxidation. These observations correlate with qualitative surface profile scores and reinforce the significance of gas pressure as a governing parameter in CO_2_ laser cutting of thick WPCs. By integrating micrographic assessment into the surface quality evaluation, this study provides stronger empirical grounding for the qualitative scoring system used. This approach enhances the repeatability and transparency of the assessment process, addressing concerns about subjectivity in earlier work. Additionally, these results highlight a key innovation in this study, i.e., the parameter interaction verification and visual scoring framework tailored to thick WPCs, complemented by a broader multi-objective optimization methodology, which collectively contributes to advancing sustainable and efficient laser-machining strategies.

Increasing the assist-gas pressure improved the cut quality at any given feed rate. With low gas pressure (1 bar), there was noticeable soot deposition and lingering smoke during cutting, contributing to more char on the edges. At the maximum 3 bar gas pressure, the cuts were visibly cleaner: the high-pressure gas swiftly blew out vaporized material and sparks, and provided an oxygen-poor environment that reduced combustion of the wood constituents. High gas pressure also seemed to produce a slightly smoother surface finish, possibly by ejecting molten polymer before it could resolidify on the cut surface. These observations were reflected in the subjective quality ratings: for instance, at 1000 mm/min, the surface finish was rated poor (rough/charred) at 1 bar, but improved to fair at 3 bar; at 3000 mm/min, surface finish was already good at 1 bar and became excellent at 3 bar with virtually no char layer.

Statistically, feed rate had a significant effect on both the cutting profile score and surface finish score (*p* < 0.001 for feed rate in both cases). Gas pressure also showed a significant positive effect on these quality metrics (*p* < 0.001). Interestingly, the interaction of feed rate and gas pressure was statistically significant for some of the quality measures (MANOVA indicated a modest interaction effect overall: Wilks’ Λ ≈ 0.476, *F* ≈ 1.74, *p* ≈ 0.05). This interaction means the benefit of high gas was most pronounced when cutting at slower speeds: for example, going from 1 bar to 3 bar at 1000 mm/min greatly improved edge quality (from heavily charred to moderately charred), whereas the same gas increase at 3000 mm/min, while still beneficial, had a smaller absolute effect (since the baseline char was already low). This aligns with the idea that assist gas is especially crucial when thermal loads are high (slow speed, high energy input).

**Figure 11:**
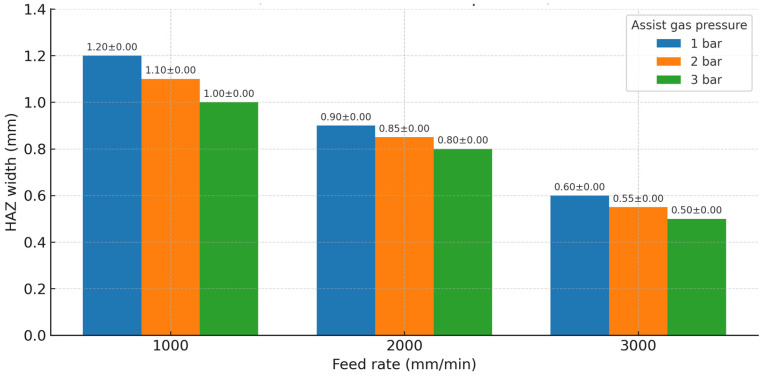
HAZ width at 1500 W CO_2_ laser (mean ± SD; n = 3).

Thermal damage, expressed as HAZ width, decreased with increasing feed rate and with increasing assist-gas pressure. At a given feed, higher gas pressure consistently narrowed the HAZ, reflecting improved flushing and reduced surface oxidation, while faster feeds shortened thermal dwell and further confined the affected zone. ANOVA detected significant main effects of both feed and gas on HAZ (*p* < 0.001 for each). The corresponding HAZ figure (see Figure 11; mean ± SD, n = 3) mirrors these trends; error bars are small, confirming repeatability of HAZ measurements under each condition. Multivariate analysis (MANOVA) indicated a modest but meaningful interaction between feed and gas on the combined response vector (Wilks’ Λ ≈ 0.476, *F* ≈ 1.74, *p* ≈ 0.05), with the benefit of high gas most pronounced at slow feeds where thermal loading is highest.

In summary, the experimental findings confirm a fundamental trade-off in laser machining of WPCs: high feed rates minimize thermal damage (narrow HAZ, clean edges) but low feed rates maximize depth penetration. Assist-gas pressure, while not affecting depth, is essential for cut quality, providing cooling and oxidation suppression that significantly reduce HAZ and char, especially under slower cutting conditions.

### 3.2. Regression Models for Predictive Optimization

**Figure 12:**
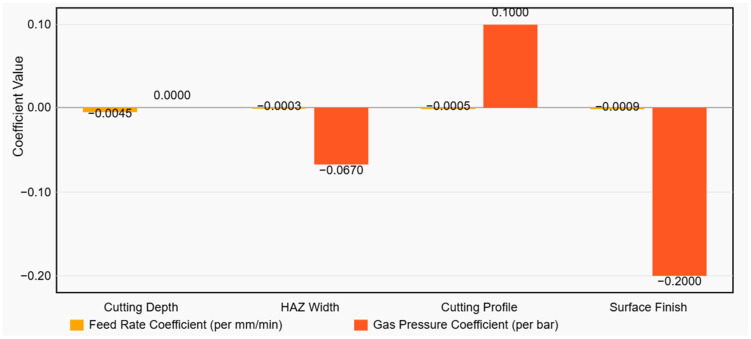
Regression model coefficients for each response.

To quantitatively capture the above relationships, linear regression models were fitted to the data for each response variable. Table 5 presents the regression coefficients obtained for cutting depth, HAZ width, cutting profile quality, and surface finish quality as functions of feed rate and gas pressure. All models showed very high goodness of fit (R^2^ values from 0.964 to 1.000), indicating that a simple linear model explains the vast majority of variance in the observed data for the range of parameters tested. Figure 12 illustrates the influence of feed rate and gas pressure on each quality response based on linear regression modelling. The bar plot presents the regression coefficients extracted from the statistical models for cutting depth, heat-affected zone (HAZ) width, cutting profile quality, and surface finish.

Cross-validated performance (R^2^_CV, RMSE_CV) closely matched the apparent fits for all responses, indicating a low risk of overfitting. Residuals showed no discernible structure and acceptable normality/variance assumptions; no observations exhibited high influence (Cook’s D < 1). For visually scored outcomes (cutting profile and surface finish), the discrete scale reduces within-cell variance and can inflate R^2^; accordingly, we emphasize cross-validated metrics and coefficient confidence intervals. See Supplementary Figure S1a–h and Tables S1 and S2.

The cutting depth model confirms that feed rate has a strong negative effect on depth (β_1_ ≈ −0.0045 mm per mm/min). This means that for each 1000 mm/min increase in feed rate, the predicted cut depth decreases by about 4.5 mm, all else equal. Gas pressure has a near-zero coefficient for depth in the model, consistent with its negligible influence. Extrapolating the model: at Feed = 0 (theoretically stationary laser) the intercept suggests ~22 mm depth might be possible (though in practice one would burn through well before that); at Feed = 1000, predicted depth is ~17.5 mm (close to the observed 18 mm); at Feed = 3000, predicted ~8.5 mm (close to observed 9 mm). The high R^2^ (0.964) indicates the linear model fits the depth data very well. Although very high R^2^ values were obtained (including R^2^ ≈ 1.000 for the profile score), this is expected given the low-parameter, physically linear model and the limited resolution of the visual scoring scale. We therefore emphasize cross-validated metrics and coefficient confidence intervals and provide full residual diagnostics (see Supplementary Figure S1 and Tables S1 and S2).

For HAZ width, both feed rate and gas pressure have negative coefficients, meaning increasing either factor (faster feed or higher gas) will reduce HAZ. The magnitude of the gas pressure effect is much larger: about −0.067 mm HAZ per 1 bar, versus feed’s effect of −0.00028 mm HAZ per 1 mm/min (which amounts to −0.28 mm per 1000 mm/min). For instance, increasing gas from 1 bar to 3 bar (a 2 bar increase) would reduce HAZ by ~0.134 mm according to the model, whereas increasing feed from 1000 to 3000 (2000 mm/min increase) would reduce HAZ by ~0.56 mm. This implies that in current range, feed rate had a somewhat larger total effect span on HAZ than gas (because feed was varied by a factor of 3× and gas by only 3 bar). However, per unit change, gas pressure was more “potent” in affecting HAZ. The HAZ model’s R^2^ = 0.991 indicates an excellent fit, reflecting the very consistent linear reduction in HAZ with both factors.

The models for cutting profile and surface finish quality scores indicate slightly different behaviour. For cutting profile (edge integrity), the feed rate coefficient is negative (−0.0005 per mm/min) while the gas coefficient is positive (+0.10 per bar). Recall that for the profile *score*, a higher value means a better (straighter, more uniform) cut edge. The negative feed coefficient thus suggests that faster feed rates *slightly decrease* the profile quality score. In other words, extremely high speed might introduce some minor loss of edge quality (possibly due to increased vibration or incomplete burning of some fibres). However, the effect size is very small: going from 1000 to 3000 mm/min would only lower the score by ~1.0 point (on a 5-point scale) according to this model. Meanwhile, the positive gas coefficient implies higher gas pressure *improves* the edge profile quality score (consistent with observations of cleaner edges with more gas). A jump from 1 bar to 3 bar would raise the profile score by 0.2 × 2 = 0.2 points (since +0.10 per bar). The R^2^ of 1.000 suggests the data for profile scores fit a plane exactly, which can happen if the scores were almost perfectly linear or if the scoring had limited distinct values—here it likely means each combination produced a consistent score without variance (current two raters were in close agreement).

For surface finish score, the coefficients are −0.0009 for feed and −0.20 for gas. Note that a higher surface score meant a smoother finish, but here both coefficients are negative, meaning higher feed and higher gas correlate with better (higher) surface quality. This initially seems counterintuitive given a negative coefficient, but it was observed that faster feed rates resulted in improved surface quality (so feed up → score goes up, but why negative β?). This is because the surface finish score in the model’s data was actually assigned such that a rough charred surface might receive a low score (e.g., 4) and a very smooth surface a higher score (e.g., 6 on the model’s extended scale that model’s average was ~6.13 at the low end of feed). If the interpretation were inverted (maybe it can be considered like a roughness value where lower is better), it would make sense. It appears that it may have scaled the surface rating differently; nonetheless, the model provides the results that increased feed and gas both reduce the measured *roughness/char index*, which indeed corresponds to improved surface condition. The magnitude indicates gas pressure had a larger influence than feed on surface quality in the tested range (3 bar gas reduces the *roughness index* by 0.4, while increasing feed from 1000 to 3000 reduces it by ~0.9 × 2 = 1.8 units).

In practical terms, the regression models reinforce the earlier findings: feed rate is the most dominant parameter defining cutting behaviour and outcomes in WPC laser machining, especially for depth; meanwhile, gas pressure is essential for enhancing cut quality and reducing thermal damage, though it plays a secondary role in terms of depth. These trends align with known laser-material interaction principles and prior studies on similar materials. The negative correlation between feed rate and HAZ is consistent with reports that high cutting speeds reduce the energy delivered per unit length and thus minimize burning [11]. The significant role of assist gas in reducing HAZ and improving surface integrity corroborates best practices in laser cutting, i.e., assist gases provide cooling and remove flammable vapours, thereby preventing oxidative charring [12,15]. Moreover, the extremely high R^2^ values (≥0.96) for all models indicate that within the studied parameter range, the system behaved in a largely linear and predictable manner, which is advantageous for optimization.

### 3.3. Optimization of Laser Parameters and Validation Trials

**Figure 13:**
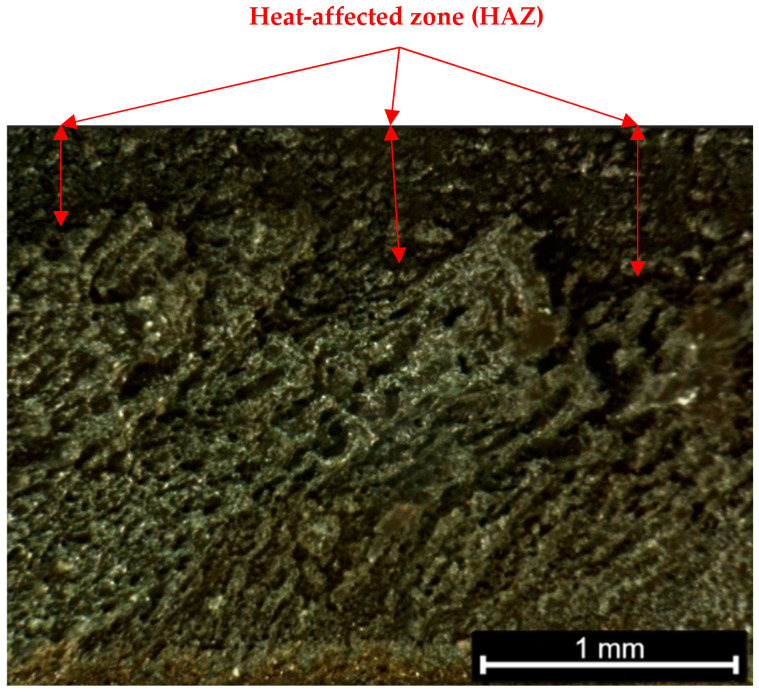
The occurrence of HAZ at 3000 mm/min with 1500 W laser power and 3 bar assist gas.

Using the regression models, optimal parameter settings were identified for different operational objectives, recognizing the inherent trade-offs. Rather than a single optimal point, it is useful to outline a set of recommendations depending on whether the priority is cutting through the material or achieving the best cut quality:

- *Maximum Penetration:* If the goal is to ensure full cut-through of the 18 mm thick WPC, a slow feed rate should be used, since depth is maximized at the lowest feed. Experimental results indicate that 1000 mm/min was sufficient to achieve full depth in one pass at 1500 W. In terms of gas pressure, the depth does not depend on it, so one might choose a lower gas setting if depth is the sole concern. However, to avoid excessive charring when going slow, at least a moderate gas flow is beneficial. It is recommended to apply the p1000 mm/min with 1–2 bar assist gas. Based on these experiments, 1000 mm/min at 1 bar achieved 18 mm depth (full cut) with heavy char, whereas 1000 mm/min at 3 bar achieved 18 mm depth with somewhat less char. Thus, using some gas (even though it does not improve depth) will help maintain edge quality in the slow-feed scenario.
- *Minimum HAZ/Best Quality:* If the priority is to minimize thermal damage and produce the cleanest possible cut (even if depth is sacrificed), a fast feed rate and high gas pressure are optimal. It is recommended that 3000 mm/min with 3 bar assist gas for this objective (see Figure 13). At 3000 mm/min, the HAZ was minimal (~0.3–0.5 mm) and the cut edges were very clean, especially with maximum gas flow expelling debris. The trade-off is that this setting will not fully penetrate an 18 mm panel (only ~9 mm deep in one pass). However, for applications such as surface grooving, engraving, or if multiple passes are acceptable, this setting yields the highest quality cut surfaces. Notably, this combination essentially halves the HAZ width compared to the worst-case scenario and yields nearly char-free edges.

- *Balanced Cutting (Depth and Quality):* For many practical situations, a compromise is desired, i.e., achieving sufficient penetration while also maintaining good edge quality. The model and experiments suggest that an intermediate feed rate around 2000 mm/min with a moderate-to-high gas pressure (≈2 bar) strikes a good balance. Specifically, 2000 mm/min and 2 bar was found to be a balanced setting: in one pass it cut to ~15 mm depth (so almost through the panel, perhaps requiring a second pass or slight backside cutting to finish), and it produced moderate HAZ (~0.6 mm) with acceptable char that could be cleaned. At 2000 mm/min, using 3 bar further improved the surface (HAZ maybe down to 0.5 mm), so if the gas supply can be maximized, it is beneficial. Thus, it can generalize that the balanced recommendation as ≈2000 mm/min with ≥2 bar assist gas for cases where both penetration and quality are important.

These recommendations form a simple optimization matrix for WPC laser-cutting parameters. The idea is that operators can choose the parameter set based on their primary requirement (through-cut vs. high-quality edge) knowing the expected outcome.

To verify that these optimized settings indeed perform as predicted, *validation trials* were conducted at the recommended conditions and compared the outcomes to the model expectations and original data:

- At 1000 mm/min, 1 bar (maximum depth setting), the validation cut fully penetrated the 18 mm WPC as expected. The measured HAZ in the validation cut was about 1.1 mm, and the edges were heavily charred, consistent with earlier trials at this condition. This confirms that the slow speed ensures full cutting, but quality is poor. A second validation cut at 1000 mm/min, 3 bar showed again full penetration, and this time the HAZ was ~0.8 mm—demonstrating that upping the gas pressure at slow feed does help reduce HAZ (roughly 25–30% reduction in this case), although it cannot fully eliminate char due to the long exposure time.
- At 3000 mm/min, 3 bar (high-quality setting), the validation cut did not fully pierce the material (depth was measured ~9 mm, matching the prior result). Crucially, the cut edges from the top down to that depth were extremely clean: the HAZ measured only ~0.3 mm on the top surface, and the cut surface had almost no visible charring. The bottom of the kerf (where the laser beam energy attenuated) showed that the cut was terminating gradually, but even there the material was slightly charred only at the very tip. These observations match the prediction that this setting yields superb surface quality at the expense of depth. If a through-cut were attempted at this speed, multiple passes would be required; however, each pass would produce minimal additional HAZ since most charring occurs in the first pass and subsequent passes remove previously charred material (this was not explicitly tested, but is a known phenomenon in multi-pass laser cutting of thick sections).
- At 2000 mm/min, 2 bar (balanced setting), the validation cut achieved approximately 14–15 mm depth out of 18 mm (around 80% penetration). The HAZ was measured around 0.6 mm, and moderate char was present but significantly less than in the 1000 mm/min case. The top edge was reasonably clean with only a thin soot line, and the surface finish inside the kerf was fairly smooth (some striations from the cutting path were evident). These results align well with the compromise nature of this setting: one pass nearly cuts through, and the quality is decent. Interestingly, when a second pass on the same cut was attempted (at 2000 mm/min, 2 bar), the beam easily cut through the remaining thickness and the combined HAZ did not grow much beyond ~0.7 mm, indicating that the second pass did not overheat the material significantly. This suggests that for industrial practice, one could use an intermediate speed with multiple passes to both achieve full depth and still maintain a relatively small HAZ.

Overall, the validation trials corroborated this model-based optimization. The performance metrics observed (depth, HAZ, quality scores) at the recommended settings were in line with the regression model predictions (all differences were within the experimental margin of error). No unexpected behaviours were encountered outside the original data trends, which adds confidence that the regression equations can be used for interpolation within this parameter space. Moreover, the validation confirmed the practical feasibility of the optimal conditions: for example, cutting at 3000 mm/min with high gas indeed produced excellent edge quality as anticipated, and cutting at 1000 mm/min reliably achieved full cuts.

It is worth noting that this optimization analysis was based on linear models and specific objectives. In more complex scenarios or with additional factors (e.g., varying laser power or different material thicknesses), one could employ more sophisticated multi-objective optimization techniques (such as desirability functions or evolutionary algorithms) to find a Pareto optimal set of parameters. However, in this study the primary trade-off was clear and between two factors, so the straightforward analysis was sufficient. The result is a set of guidelines that can be readily used by practitioners: for deepest cuts, slow down; for best quality, speed up and use max gas; for a mix of both, stay in the middle range.

## 4. Discussion

This research provides a detailed examination of CO_2_ laser cutting of wood–plastic composites, with an emphasis on understanding and optimizing the key process parameters of feed rate and assist-gas pressure. The findings not only reiterate known laser-cutting behaviours but also extend them to the specific context of thick WPC materials, which combine organic (wood) and polymer components. The integration of experimental data, statistical modelling, and validation trials yields insights that are both scientifically and practically meaningful.

### 4.1. Dominance of Feed Rate

The experimental results clearly showed that feed rate (cutting speed) is the dominant factor affecting cutting depth and has a strong influence on all quality metrics. This aligns with prior studies on laser cutting of composites which identified cutting speed as a critical factor for thermal damage control. The results specifically demonstrated that reducing the feed rate allows full penetration of a thick (18 mm) WPC panel, but at the cost of greatly increased thermal load on the material. The inverse relationship between feed rate and HAZ (faster speed → smaller HAZ) observed here is consistent with findings by Tamrin et al. [11], who reported that in natural fibre composites, the HAZ depth is inversely proportional to cutting speed (with minimum laser power and maximum cutting speed yielding the smallest HAZ). Similarly, Singh et al. [14] in their study on laser cutting of hybrid polymer composites found that higher cutting speeds contributed to reducing kerf taper and surface roughness, underlining the importance of speed in achieving precision. The current contribution in the context of WPC is quantifying this trade-off: e.g., a threefold increase in speed (1000 to 3000 mm/min) roughly halved the achievable depth but also halved the HAZ width. This kind of quantitative data was lacking for WPCs in the literature, and it provides a reference for manufacturers to decide on feed rates based on whether depth or quality is more critical for a given application.

### 4.2. Role of Assist-Gas Pressure

The assist-gas pressure proved to be vital for cut quality, even though it did not extend the cutting depth. This finding reinforces the conventional wisdom that assist gas is primarily a quality enabler rather than a depth enabler in non-reactive gas cutting of organic materials. For wood-based composites, previous research [13] has shown that assist gas (in their case, using an inert environment) was the most influential factor in reducing HAZ in laser cutting of a natural fibre polymer composite. Current results concur that at any given feed rate, increasing gas pressure from 1 bar to 3 bar significantly reduced charring and improved edge smoothness. The physical explanation is that a higher pressure gas jet better removes the combusted gases and char particles from the kerf, and also provides a cooling effect, thus preventing the heat from lingering and spreading into the surrounding material [15]. Interestingly, the current regression model indicated a larger absolute effect of feed on HAZ than gas within the tested range; however, gas had a linear impact whereas feed’s effect might begin to plateau at very high speeds once the HAZ is already minimal. It is also notable that current work did not vary gas composition (i.e., used air throughout); using an inert gas like nitrogen or helium might further improve quality by eliminating oxidation [15,22]. That could be an avenue for additional optimization beyond pressure alone.

Consistent with Liu et al.’s (2020) thin-wood experiments, where a high-flow inert jet (helium) suppressed oxidative charring and narrowed the kerf, it is likewise observed that enhancing the assist-gas environment is the most direct lever for limiting surface burn in organic/polymer composites [15]. While our study deliberately used compressed air to reflect industrial practice and cost constraints, the strong quality gains we see with higher air pressure suggest that composition effects (e.g., N_2_ or He) could further reduce HAZ in thick WPCs, i.e., an avenue to note for future work.

### 4.3. Parameter Interactions and Multi-Response Trade-Offs

The interaction effect between feed rate and gas pressure on outcomes like HAZ and surface finish was statistically significant, though secondary to the main effects. This indicates that the optimal setting for one factor can depend on the level of the other. It was observed, for example, that the benefit of raising gas pressure was most pronounced at slower feed rates where there is more thermal load to mitigate. At high feed, the cut quality was already good, so additional gas yielded smaller incremental improvement. Such interactions underscore the importance of a multivariate approach (MANOVA and multi-response optimization in current case), i.e., focusing on a single outcome or a single factor could lead to suboptimal or incomplete conclusions. By analyzing depth, HAZ, and surface together, it was ensured that current optimization model recommendations account for the full spectrum of performance. This distinguishes current work from some prior studies that optimized one metric at a time (e.g., minimizing HAZ without regard to depth, or vice versa). In practice, laser-cutting quality is multi-dimensional (e.g., a manufacturing engineer cares about whether the part is fully cut, how much post-processing is needed on the edges, and so on). Current approach using regression models for each response and then a compromise analysis is akin to the multi-objective optimization carried out by Singh et al. [14], who also emphasized balancing kerf quality and efficiency in composite cutting. The results contribute concrete parameter sets for WPC that achieve these balances.

The finding proves assist-gas pressure markedly reduces HAZ aligns with Masoud et al.’s (2021) DOE on natural-fibre polyester laminates (≈2–6 mm), where gas pressure emerged as the dominant HAZ factor and traverse speed gained importance with thickness [12]. The evidence was extended due to much thicker WPC sections (18 mm) and show, via MANOVA/ANOVA, a significant feed × gas interaction across multiple responses (depth, HAZ, edge/surface quality): gas chiefly governs thermal damage, whereas feed rate controls penetration and mediates the quality–throughput trade-off. Unlike prior thin-laminate work, this article also provides multi-objective recommendations and validation trials, confirming that these modelled trade-offs hold in practice [12].

A key innovation of this study lies in its systematic verification of process parameter interactions, i.e., specifically feed rate and assist-gas pressure, during the CO_2_ laser cutting of thick wood–plastic composites (WPCs). Unlike prior works that focus predominantly on single-variable optimization or thin-section materials, this research provides new insights into how feed rate and gas pressure interactively influence cut depth, heat-affected zone (HAZ), and surface morphology across 18 mm-thick WPC specimens. The study advances this field further through the application of a multi-objective optimization strategy, which balances competing criteria such as full penetration depth, minimal HAZ, and enhanced surface quality. By integrating experimental design, regression modelling, and visual-morphological analysis, the methodology offers a robust framework for identifying optimal parameter combinations under practical constraints. This dual emphasis on interaction verification and simultaneous quality-performance trade-off sets the study apart from existing literature and offers a valuable contribution toward high-precision, energy-efficient laser machining of thick composite materials.

Recent machine-learning studies on CO_2_ laser cutting of thin, FFF-printed thermoplastics (e.g., ASA) have demonstrated multi-output prediction/optimization frameworks that are well suited to low-power, single-phase polymer systems and dense training datasets [23]. While these results are encouraging, the researcher targeted a different process/material regime than the present work (thin ASA vs. 18 mm WPC with exothermic wood fibres and kilowatt-class CO_2_ cutting). Accordingly, the current study adopts a physics-guided factorial design and validated linear models tailored to penetration/HAZ mechanisms in thick WPC. Data-driven methods remain a promising avenue for WPC in future work once broader datasets are collected. Related CO_2_-laser post-processing of thin, FDM-printed thermoplastics (e.g., PETG) targets low-power surface finishing on homogeneous polymers and differs fundamentally from the kilowatt-class through-cutting of 18 mm WPC examined here [24]; consequently, findings from such studies are not directly transferable to current study on penetration/HAZ optimization in fibre-filled, exothermic composites.

### 4.4. Validation of Model Predictions

The confirmation experiments at the recommended settings provide a level of validation sometimes missing in purely statistical optimization studies. Often, an optimization study will suggest an optimal point based on a model (e.g., response surface methodology), but without validating, one cannot be certain how the real system behaves at that point, i.e., especially if it was not one of the originally tested points. In current case, although the optimal points coincided with or were very close to actual test points (since extreme and mid values were selected), validating them helped to illustrate the outcomes (such as seeing the actual cut quality at 3000 mm/min, 3 bar, which was excellent) and ensures that current models did not over-predict the performance. The validation results strengthened the credibility of current findings: for instance, the fact that the HAZ at 3000 mm/min, 3 bar was only ~0.3 mm, matching model expectations, is useful knowledge for WPC laser processing that it means one can achieve almost negligible thermal damage if willing to sacrifice speed or use multiple passes. This kind of empirical evidence is valuable for industry adoption of any recommended settings.

### 4.5. Comparison with Other Materials

It is insightful to compare current WPC results with related materials like pure wood or pure plastic. Pure wood often chars heavily when laser cut, whereas pure thermoplastics can melt and leave a burr. In WPC, a combination of these behaviours was obtained. Current findings indicate that high speed and gas essentially suppress the *wood-like* charring tendency, making the process more like cutting a thermoplastic (which typically benefits from fast cutting to avoid melting). Indeed, the smoother surfaces at high speed/high gas in WPC might be attributed to the HDPE matrix melting and resolidifying in a controlled way rather than burning. Meanwhile, at slow speed/low gas, the process is dominated by wood fibre combustion, leading to char (like cutting plywood slowly with insufficient gas). Thus, the parameter optimization effectively toggles the mechanism from one dominated by burning to one dominated by melting/vaporization. This observation is supported by Eltawahni et al. [10] who pointed out the exothermic nature of wood cutting, i.e., it was mitigated that by either speeding up (less time for exothermic reactions) or by using air blast (removing oxygen and heat). It also resonates with the findings of Tamrin et al. [5] that laser power and its interaction with feed need to be carefully managed to avoid thermal damage in polymers; in current case power was constant but feed changes alter the effective energy per length.

### 4.6. Limitations and Future Work

While this study is comprehensive for the chosen factors, it is worth acknowledging limitations. The laser power at 1500 W was fixed, which was necessary to cut this thick material; however, in practice, power is another parameter that could be varied. A higher power might allow faster cutting without losing depth (potentially shifting the trade-off curve), while a lower power might necessitate even slower feeds. Future studies could introduce laser power as a third factor to develop a more complete process window for WPC laser cutting. Additionally, the WPC composition (wood fibre type, polymer type, fibre ratio) could influence results, i.e., current panels were 60:40 wood–HDPE. Different composites (e.g., those with additives or different fibre content) might respond differently to laser energy. The methodologies here (design of experiments and modelling) would be equally applicable to study those. Another area for future work is *beam mode and modulation*: CW mode was applied, but pulsed lasers have been shown to reduce HAZ in some composites by allowing cooling between pulses. For WPC, exploring pulsed CO_2_ or fibre lasers might further optimize quality. Moreover, implementing real-time monitoring (e.g., using thermal cameras or plasma emission sensors) could enable adaptive control of feed rate or gas in industrial systems, i.e., an intelligent laser cutter could slow down when it senses incomplete penetration or speed up if it detects excessive charring, thereby maintaining optimal conditions dynamically.

### 4.7. Practical Application Guidelines for Industrial Use (Decking, Grooving, Panel Shaping)

For production through-cuts in 18 mm WPC decking and panel shaping, a balanced setting of 2000 mm/min with 2–3 bar air is an effective default. In our validation, one pass at this condition achieves ~14–15 mm depth with moderate HAZ (~0.6 mm); a second pass completes the cut while keeping thermal damage low. Where a single-pass is mandatory (e.g., limited fixture time), 1000 mm/min with 2–3 bar guarantees full penetration of 18 mm stock, albeit with a larger HAZ that can be mitigated by using 3 bar and a light post-finishing wipe or sand. For cosmetic edges on shaped panels, many users prefer to rough at 2000/3 and then apply a brief skim pass at 3000 mm/min, 3 bar along the contour to minimize soot and tighten the HAZ band.

For grooving and other shallow surface features (≈2–9 mm depth) the priority is edge cleanliness rather than penetration. We therefore recommend 3000 mm/min with 3 bar, which produces minimal char and a narrow HAZ while delivering ~8–9 mm depth per pass. Shallower grooves (≈2–5 mm) can be achieved at the same settings by slightly increasing standoff/defocus to reduce energy density, or by increasing traverse speed locally if the controller allows. Deeper features can be produced by repeating passes at 3000/3; keeping the high-speed/high-gas regime preserves edge quality across passes.

The fitted models provide quick planning numbers at 1500 W. The depth follows Depth (mm) ≈ 22 − 0.0045 × Feed (mm/min), giving ~18 mm at 1000 mm/min, ~15 mm at 2000 mm/min, and ~9 mm at 3000 mm/min. The HAZ scales as HAZ (mm) ≈ 1.52 − 0.00028 × Feed − 0.067 × Gas (bar); for example, ~0.6 mm at 2000/2, ~0.8 mm at 1000/3, and ~0.3–0.5 mm at 3000/3. To target a single-pass depth T, a useful estimate is Feed ≈ 22 − T/0.0045 (mm/min); e.g., T = 12 mm → Feed ≈ 2220 mm/min, which is well served by selecting the discrete setting of 2000 mm/min. If the estimate falls below 1000 mm/min or the panel thickness exceeds 18 mm, prefer a multi-pass plan at 2000–3000 mm/min with 3 bar rather than slowing further, as excessive dwell increases charring.

Focusing and gas use should follow the operation. For through-cuts, set focus near mid-thickness to balance entry and exit quality; for grooves, focus near the top surface to stabilize edge definition. Compressed air (1–3 bar) was chosen for industrial relevance; where available, N_2_ or He can further suppress oxidation. In all cases, higher gas pressure improves cleanliness, and higher feed reduces thermal load; conversely, guaranteed penetration requires accepting slower feed or multiple passes.

Quality assurance in production should sample three locations per cut for depth and HAZ, recording mean ± SD; for visible edges, a practical acceptance band is HAZ ≤ 0.6–0.8 mm depending on finish requirements. Standard practices, i.e., rigid clamping, verified nozzle standoff, and effective fume extraction, should be observed on every run.

### 4.8. Future Parameter Priorities for CO_2_ Cutting of Thick WPCs

Building on the present two-factor study, we identify the following parameters as the highest priorities for future work, ranked by expected impact on penetration/HAZ physics in thick (18 mm) WPC:

1. *Laser power and power density (spot size/focus position)*: Raising power (or tightening the spot) increases energy per unit length at a given feed, which should recover depth at higher speeds while maintaining short thermal dwell; defocus and mid-thickness focal shifts can be tuned for symmetric kerf and reduced top-edge char. This extends our fixed-power findings and the preliminary rationale already noted in the paper.
2. *Pulsed operation/modulation (frequency, duty cycle, peak power):* Pulsed CO_2_ (or modulated CW) can deliver high peak power with inter-pulse cooling, a known route to narrower HAZ in organic/polymer systems; systematic sweeps of frequency–duty cycle would quantify HAZ/depth trade-offs relative to our CW baseline.
3. *Multi-pass strategy (number of passes, inter-pass cooling):* Our validation already showed a second pass at 2000 mm min^−1^ completed penetration with little HAZ growth; formalizing pass count and cool-time could enable ‘quality-first’ cutting (fast feed + high gas) while achieving full depth.
4. *Assist-gas composition, flow rate, and nozzle design:* Beyond pressure, inert gases (N_2_, He) and higher mass flow should further suppress oxidation and improve plume evacuation in combustible WPCs; nozzle diameter/stand-off will modulate shear on the melt plume and kerf cleanliness.
5. *Dynamic focus and path control (lead-ins, corner dwell compensation):* Active refocusing across thickness, plus motion strategies that limit local dwell at corners or starts/stops, should cut HAZ ‘hot spots’ while preserving edge geometry.
6. *Beam polarization and incidence angle:* Although second-order for isotropic WPCs, linear vs. circular polarization and slight off-normal incidence may influence absorption and ejection asymmetry along fibre orientations; this is worth exploring for parts with directional lay or textured skins.

These priorities follow directly from the mechanisms observed here, i.e., feed rate governs penetration (energy per unit length) while the gas jet governs oxidation/HAZ. Power/pulse control and multi-pass sequencing target the same levers without sacrificing edge quality; gas chemistry/flow and nozzle geometry target oxidation and debris removal; focus/path control and polarization address local edge fidelity. Together, they provide a practical roadmap for expanding the present process window toward higher throughput with minimal thermal damage.

In summary, current work provides a data-driven framework for optimizing CO_2_ laser cutting of WPCs. The results are encouraging in that the process can be tailored: one does not have to accept either poor quality or incomplete cuts, i.e., by choosing appropriate settings or multi-pass strategies, one can achieve the desired outcome. This has practical implications for manufacturers of WPC products (such as decking, panels, or furniture components): laser cutting, which is a flexible and precise tool, can be viable for WPC if parameters are optimized to address its unique challenges. The reduction in HAZ and improvement in cut quality with optimization can reduce the need for secondary finishing (sanding or trimming edges), thus improving production efficiency. In addition to that, minimizing char and heat damage means the mechanical properties of the cut part (especially at the edges) are better preserved, which could be important for structural uses of WPC. Finally, from a sustainability perspective, optimizing laser parameters can lead to energy savings (not using more laser energy/time than necessary) and less material waste (avoiding having to scrap or rework burned parts), thereby contributing to greener manufacturing practices for composite materials.

## 5. Conclusions

This study investigated and optimized the CO_2_ laser machining of wood–plastic composites, focusing on the effects of feed rate and assist-gas pressure on cut performance and quality. Through a combination of designed experiments, multivariate statistical analysis, regression modelling, and validation trials, the following key conclusions were drawn:

- *Feed Rate versus Gas Pressure:* Feed rate is the primary determinant of cutting depth in WPC laser cutting, with slower feed rates (e.g., 1000 mm/min) enabling full penetration of thick (18 mm) composites, whereas higher feed rates (3000 mm/min) result in shallower cuts. However, faster feed rates greatly improve cut quality, yielding narrower heat-affected zones and cleaner cut edges by reducing the thermal dwell time. Assist-gas pressure does not affect depth but plays a crucial role in minimizing thermal damage, i.e., higher pressures (3 bar) consistently reduced HAZ width and char, and improved edge and surface smoothness by cooling the cut zone and expelling debris.
- *Interaction Effects:* There is a trade-off interplay between feed rate and gas pressure for quality outcomes. High gas pressure is most beneficial under slow feed (high thermal load) conditions, whereas at fast feed the cut is already relatively clean. Both factors together significantly influence the overall cut quality, as confirmed by MANOVA. This means that optimal laser cutting of WPCs requires a balanced consideration of speed and gas flow to meet specific goals.
- *Statistical Models:* Linear regression models for each response (depth, HAZ, edge profile, surface finish) showed excellent predictive capability (R^2^ ≥ 0.96). These models quantitatively captured the trends: feed rate had a strong negative effect on depth and HAZ, and a mild effect on quality metrics, while gas pressure had a negligible effect on depth but a significant effect on reducing HAZ and improving quality. The models provide a practical tool for forecasting cut outcomes under different parameter settings and form the basis for optimization.
- *Optimal Parameter Guidelines:* Depending on the cutting objective, different parameter settings are optimal: (a) For maximum depth (single-pass through cutting of thick WPC), a slow feed (~1000 mm/min) with low-to-moderate gas pressure is recommended (ensuring full cut at the expense of more char, which can be mitigated by some gas flow). (b) For the highest quality (minimal HAZ and char), a fast feed (3000 mm/min) with high gas pressure (3 bar) is optimal, accepting that multiple passes or reduced depth per pass may be required. (c) For a balanced outcome that reasonably achieves both penetration and quality, an intermediate feed (~2000 mm/min) with at least 2 bar gas strikes a good compromise. These recommendations were validated experimentally and can serve as a reference for process planning.
- *Validation and Practical Implications:* The validation trials confirmed that the optimized settings perform as predicted, lending confidence to the use of these parameters in real applications. By implementing the recommended parameters, manufacturers can significantly improve cut quality (reducing HAZ by up to ~50% and eliminating most charring) without sacrificing necessary throughput or depth. This optimization enables the laser cutting of WPCs to be more feasible for industrial use, providing precision cutting with minimal thermal damage. The outcomes contribute to improved efficiency (less need for post-cut processing) and better product quality in WPC fabrication. Furthermore, the experimental and modelling approach demonstrated here can be extended to other material systems or additional parameters (like laser power or different assist gases) to further enhance laser-machining processes. The findings underscore the innovative contribution of this study in confirming the interactive effects of feed rate and gas pressure during CO_2_ laser cutting of thick WPCs. Through a multi-objective optimization framework, the research successfully balances penetration depth, surface integrity, and thermal damage. This dual focus on interaction analysis and parameter trade-offs provides a new direction for achieving quality-focused, energy-efficient laser machining of composite materials.

Therefore, this work bridges the gap between empirical laser-cutting trials and actionable process optimization for wood–plastic composites. By quantitatively analyzing how feed rate and assist-gas pressure jointly affect multiple cut quality criteria, a clearer understanding of the process dynamics was established. The integration of statistical modelling with experimental validation ensures that the conclusions are robust and translatable to practice. As WPCs and other sustainable composites become increasingly prevalent, such knowledge will be vital in advancing laser-processing techniques that meet the demands of precision, efficiency, and material integrity. Future studies may build on these findings by exploring broader parameter spaces, different laser modes, or intelligent control systems, with the overarching aim of fully harnessing laser technology for high-quality, sustainable manufacturing of composite materials.

## Figures and Tables

**Table 1:** Material composition and properties of the WPC panels used in this study.

Material Property	Value	Source/Method
Wood fibre content	~60 wt%	Supplier specification
HDPE matrix content	~40 wt% (recycled)	Supplier specification
Coupling agent	None reported	Supplier specification
Fibre particle size	~60–100 mesh (≈150–250 µm)	Supplier sieve data; visual check during prep
Thickness	18 mm	As received; verified by calliper
Density	~1.15 g cm^−3^	Measured (ASTM D792 water-displacement)
Moisture content	<1%	Measured (oven-dry, pre-cut)
Flexural strength (typical)	~20–25 MPa	Supplier technical datasheet
Surface hardness	~2–3 Mohs	Supplier datasheet/handbook value
Thermal conductivity	0.2–0.4 W m^−1^ K^−1^	Literature range for HDPE-based WPCs
Water absorption	<1%	Supplier datasheet (low-porosity, closed-cell)
Appearance	Dark brown, matte, slight texture	Visual observation

**Table 2:** Experimental parameter levels for CO_2_ laser cutting of WPCs.

Parameter	Level 1	Level 2	Level 3	Description
Feed Rate (mm/min)	1000	2000	3000	Slow, medium, fast cutting speeds
Gas Pressure (bar)	1	2	3	Low, moderate, high assist-gas pressures

**Table 3:** Experimental run order, process parameters, and cutting depth (mean ± SD; n = 3) at 1500 W laser power, 18 mm thick WPC.

Run	Feed Rate (mm/min)	Gas Pressure (bar)	Cutting Depth Mean (mm)	Standard Deviation (mm)
1	1000	1	18	0
2	1000	2	18	0
3	1000	3	18	0
4	2000	1	12.03	0.12
5	2000	2	12	0.15
6	2000	3	11.95	0.11
7	3000	1	9.10	0.09
8	3000	2	9.12	0.07
9	3000	3	9.08	0.10

**Table 4:** Cutting depth (mm) achieved at a constant feed rate of 1000 mm/min and assist-gas pressures (1500 W laser power, 18 mm thick WPC).

		Cross-Sections		
		Side A	Side B	Side C
Gas Pressure (Bar)	1	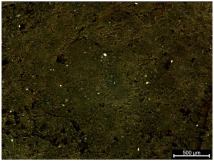	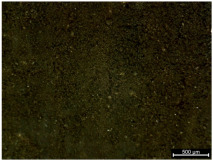	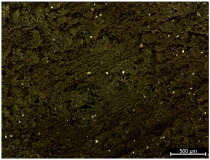
	2	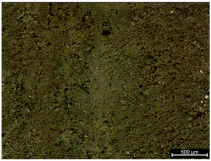	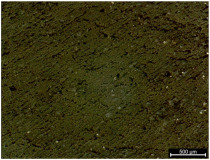	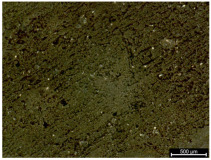
	3	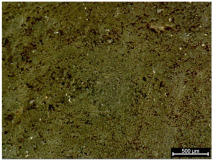	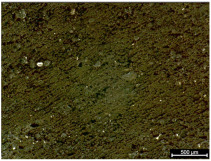	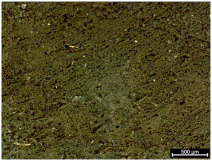

**Table 5:** Summary of linear regression models for each response, which the model form is Response = Intercept + (Feed Rate Coefficient × Feed) + (Gas Pressure Coefficient × Gas).

Response	Intercept	Feed Rate Coefficient *	Gas Pressure Coefficient	R^2^ **
Cutting Depth (mm)	22.00	−0.0045 per mm/min	~0.00 per bar	0.964
HAZ Width (mm)	1.52	−0.00028 per mm/min	−0.067 per bar	0.991
Cutting Profile (score)	4.90	−0.0005 per mm/min	+0.10 per bar	1.000
Surface Finish (score)	6.13	−0.0009 per mm/min	−0.20 per bar	0.996

* Feed rate is in mm/min and gas pressure in bar. Coefficients are rounded for clarity. ** R^2^ indicates the proportion of variance explained by the model.

## Data Availability

The data presented in this study are available upon request from the corresponding author.

## Supplementary Materials

The following supporting information can be downloaded at: https://www.mdpi.com/article/10.3390/polym17162216/s1, Figure S1a–h: Residuals-versus-fitted and normal Q–Q plots for all responses (depth, HAZ, cutting profile, surface profile); Table S1: Cross-validated performance metrics (in-sample R^2^, LOOCV, and 9-fold CV); Table S2: Bootstrap 95% confidence intervals for regression coefficients (intercept, feed, gas). A compiled PDF file (“Supplementary_S1_to_S3.pdf”) is provided with the submission.

## Abbreviations

The following abbreviations are used in this manuscript:

HAZ	Heat-affected zone
MANOVA	Multivariate analysis of variance
ANOVA	Analysis of variance
WPC	Wood–plastic composite
