# Supplementary material for: Optimization and Validation of CO2 Laser-Machining Parameters for Wood–Plastic Composites (WPCs)
Source: Polymers (Basel). 2025 Aug 13;17(16):2216. doi: 10.3390/polym17162216 (PMC12389371; doi:10.3390/polym17162216)
Supplement: Supplementary file 1 [file polymers-17-02216-s001.zip › polymers-3810689-supplementary.pdf]

Supplementary Information

Diagnostics, Cross-Validation, and Coefficient Uncertainty

Contents:

• Figure S1a–S1h: Residuals vs Fitted and Normal Q–Q plots for Depth, HAZ, Cutting Profile, Surface Profile

• Table S1: Cross-validated performance metrics (in-sample R^2, LOOCV, 9-fold CV)

• Table S2: Bootstrap 95% confidence intervals for regression coefficients (Intercept, Feed, Gas)

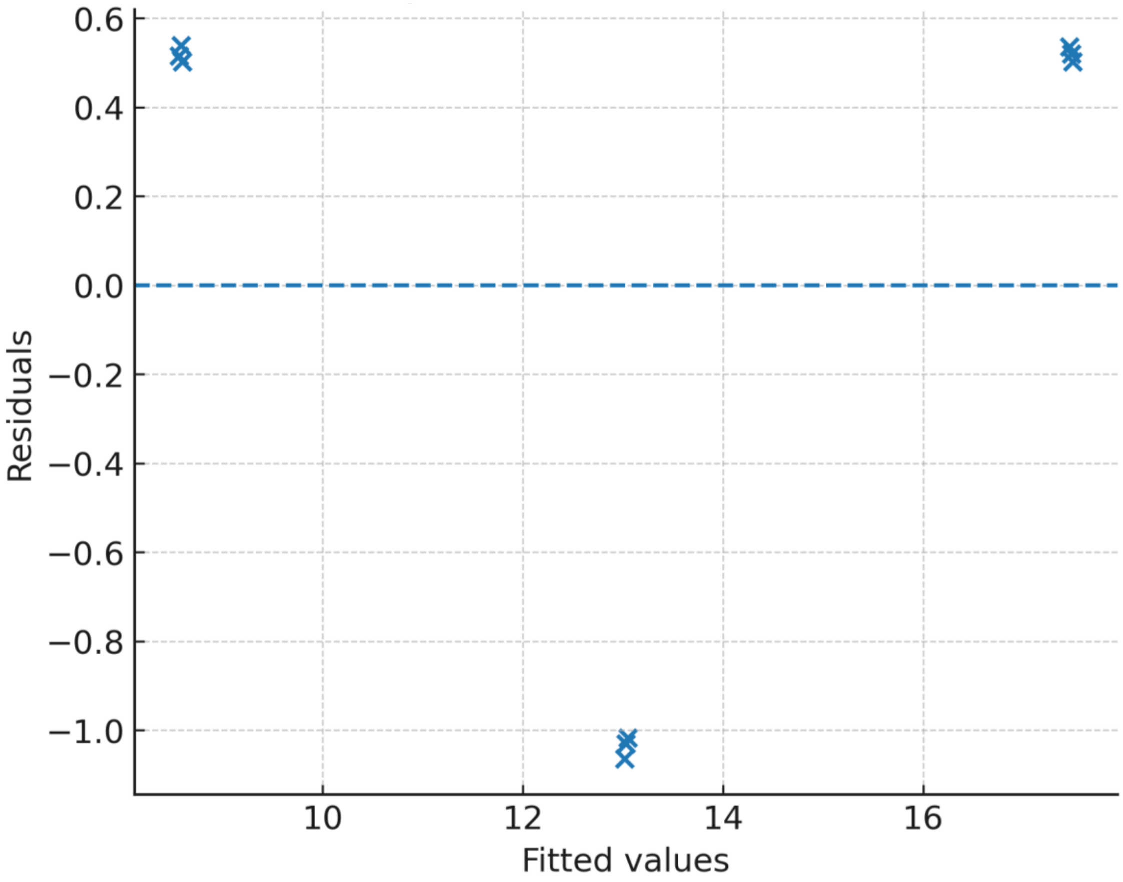

Figure S1a. Depth - Residuals vs Fitted

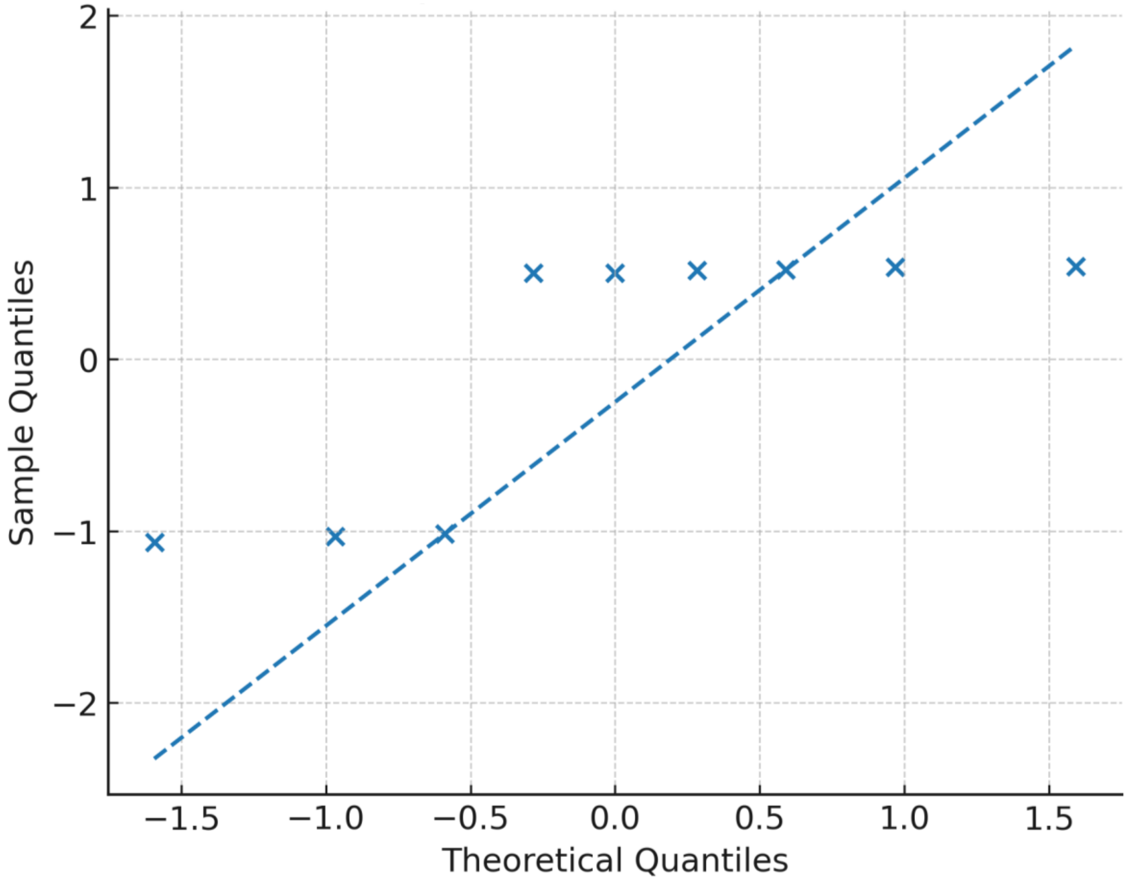

Figure S1b. Depth - Normal Q–Q plot

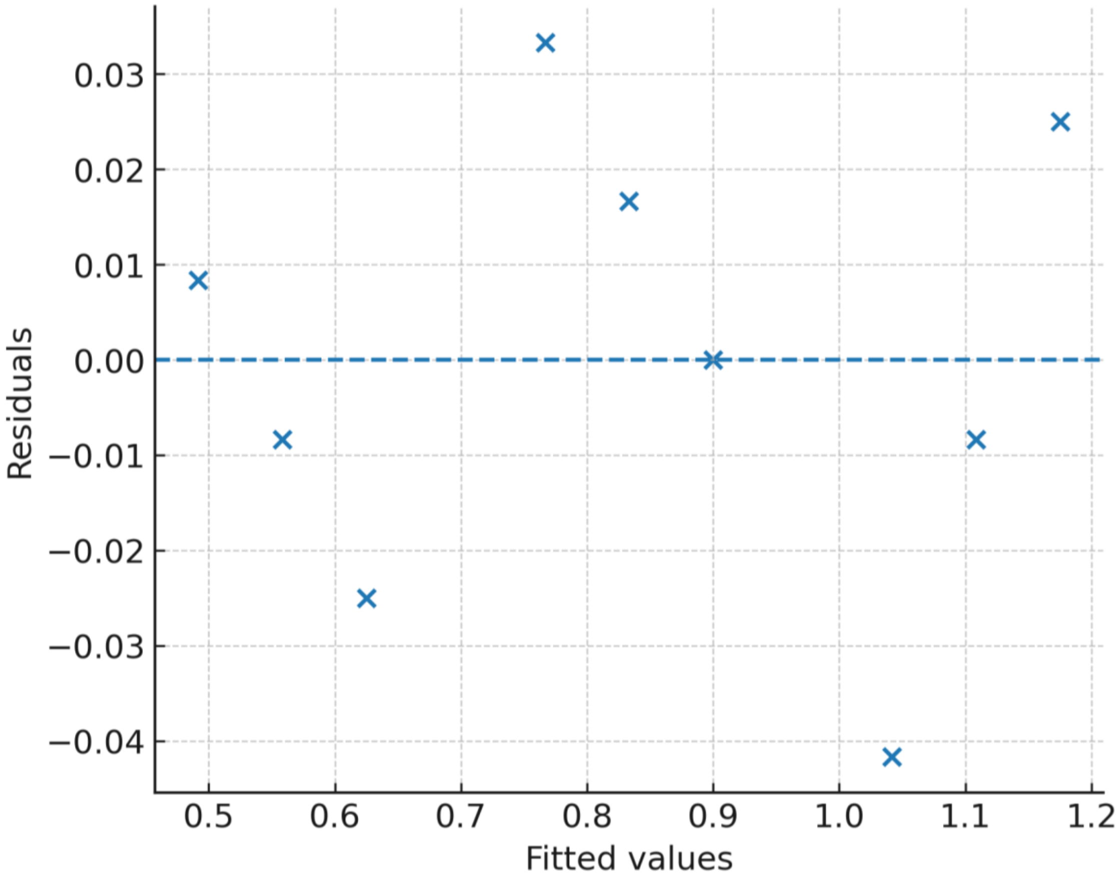

Figure S1c. HAZ - Residuals vs Fitted

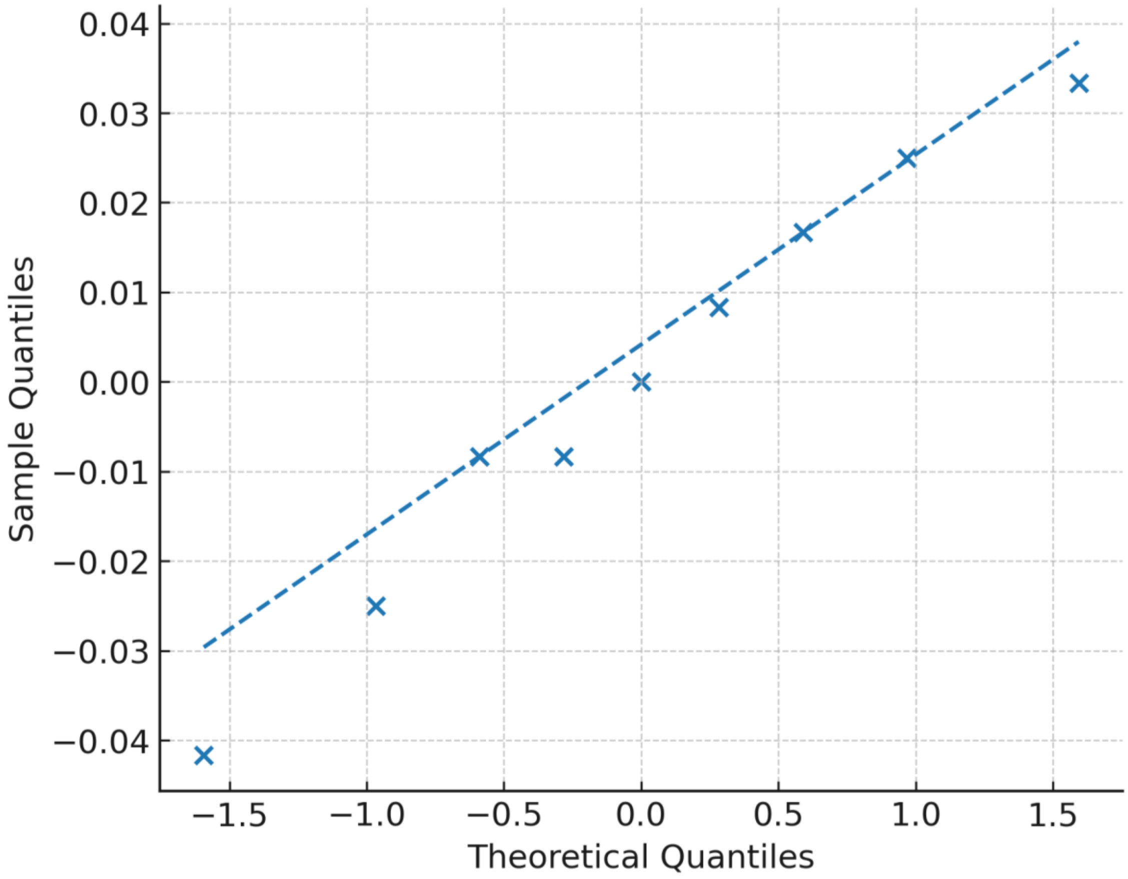

Figure S1d. HAZ - Normal Q–Q plot

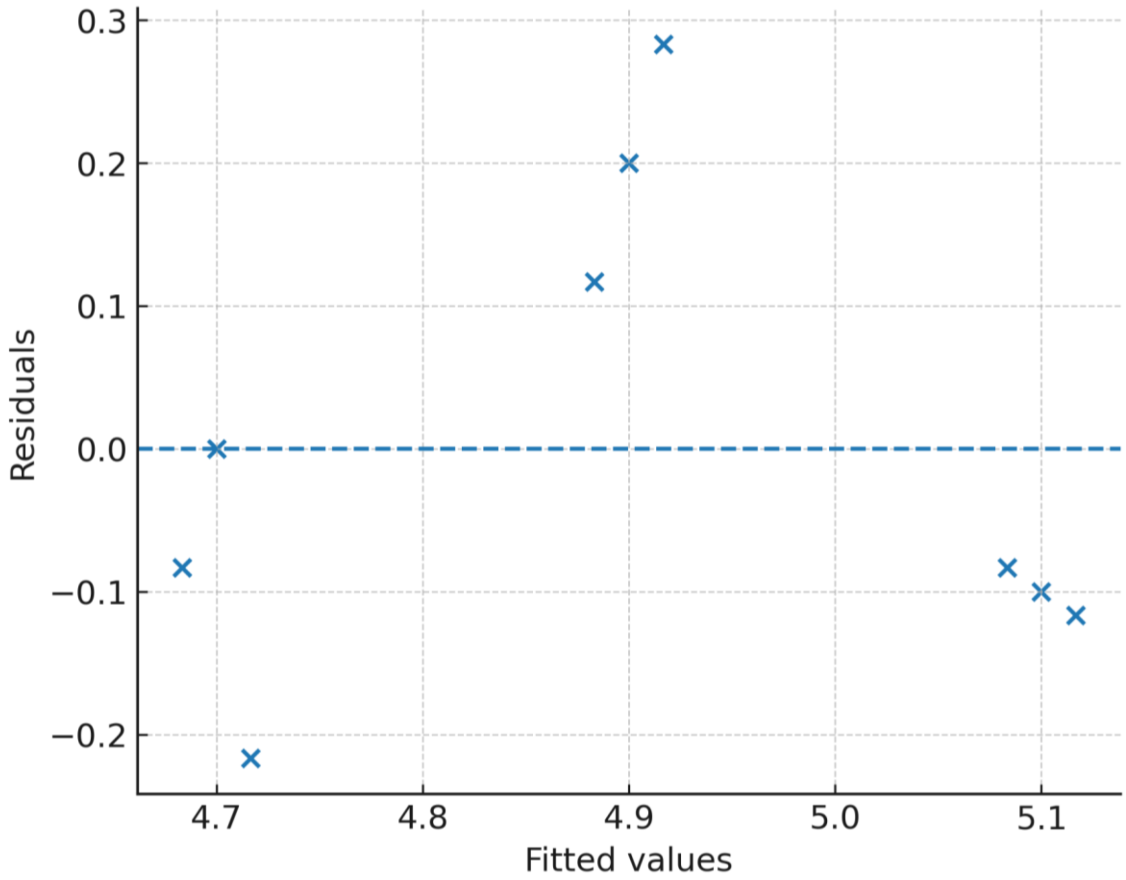

Figure S1e. Cutting Profile - Residuals vs Fitted

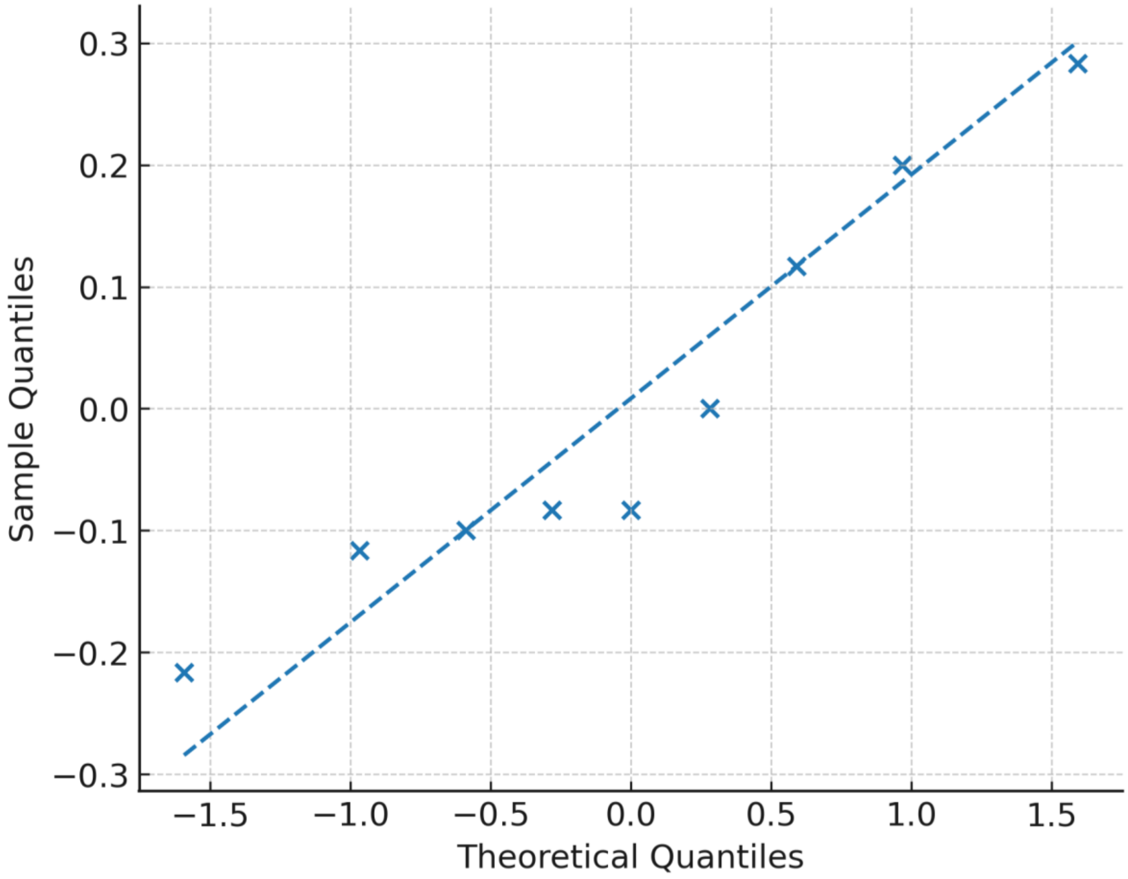

Figure S1f. Cutting Profile - Normal Q–Q plot

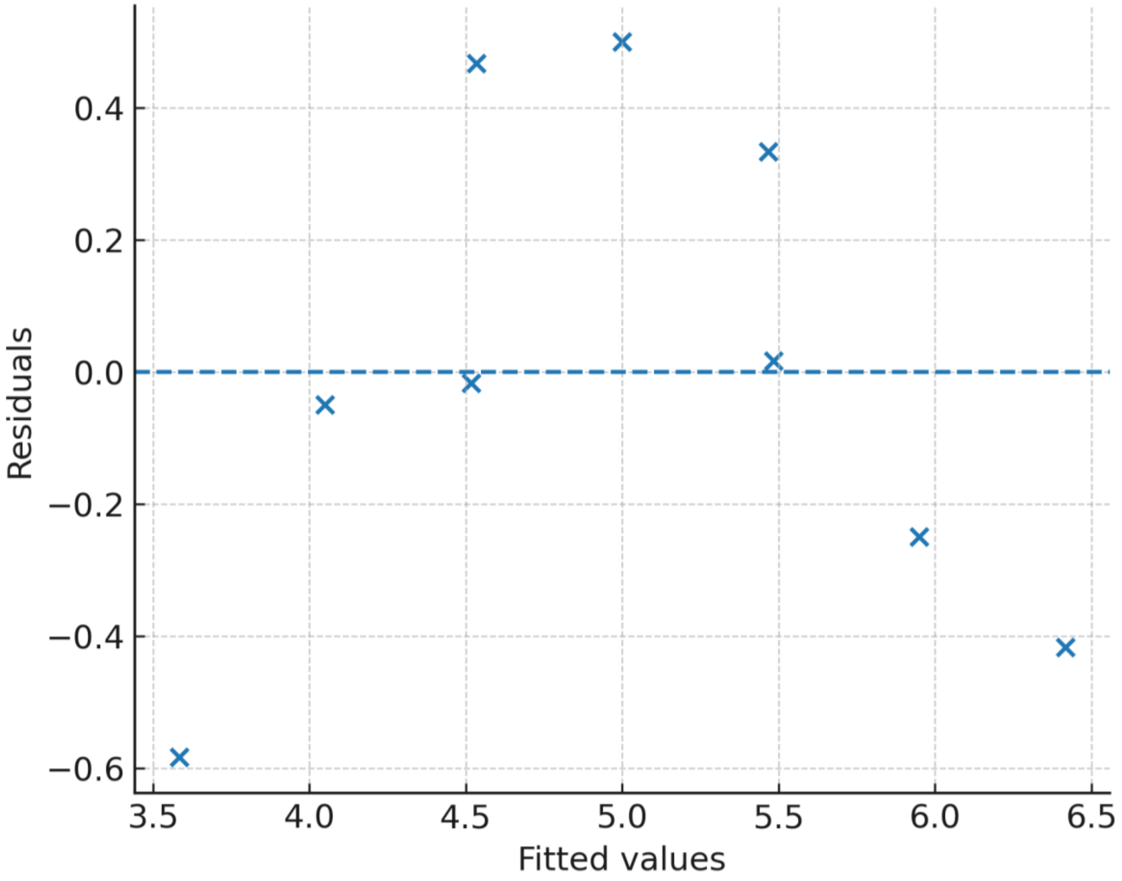

Figure S1g. Surface Profile - Residuals vs Fitted

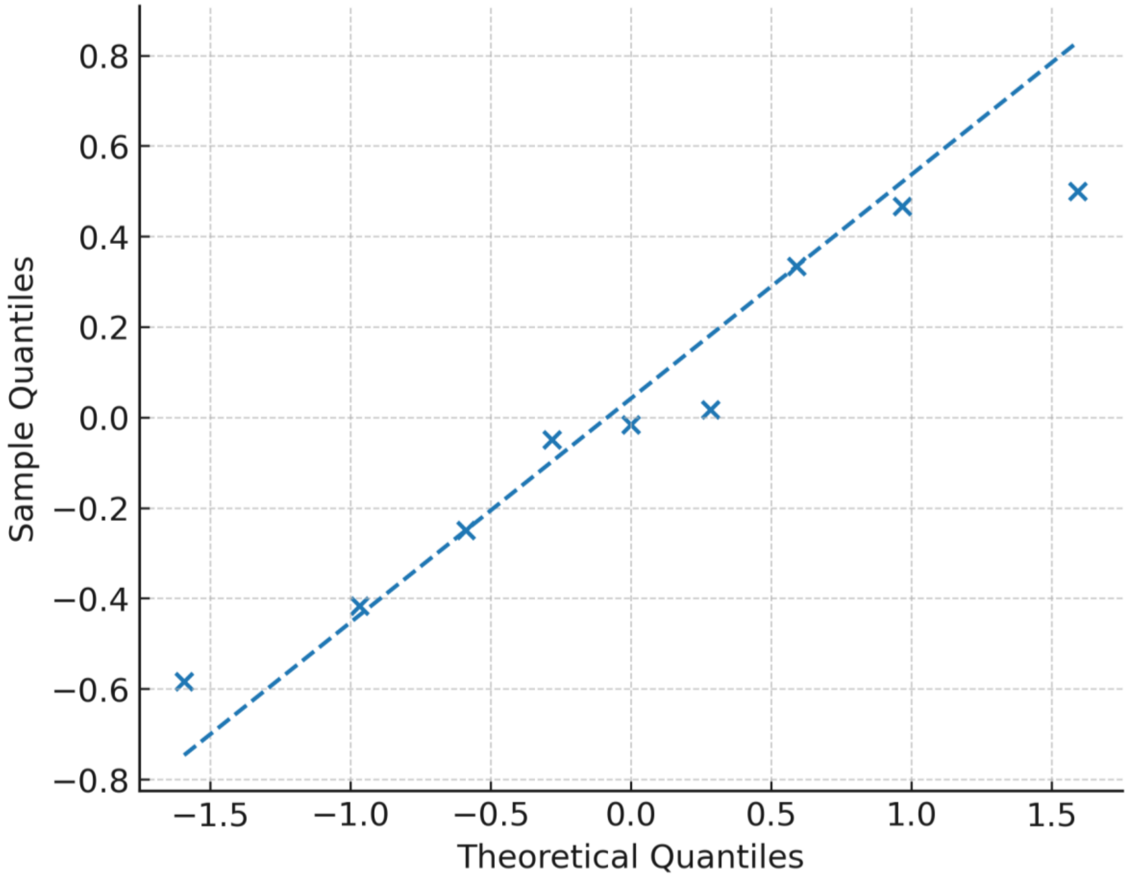

Figure S1h. Surface Profile - Normal Q–Q plot

Table S1. Cross-validated performance metrics

Response	R2_in_sample	R2_LOOCV	RMSE_LOOCV	MAE_LOOCV	R2_KFold9	RMSE_KFold9	MAE_KFold9
**Depth**	0.9608	0.9191	1.0542	1.0231	0.9191	1.0542	1.0231
**HAZ**	0.9905	0.9739	0.0375	0.0298	0.9739	0.0375	0.0298
**CutProfile**	0.5254	-0.0674	0.2336	0.2019	-0.0674	0.2336	0.2019
**SurfaceProfile**	0.853	0.6525	0.5516	0.4384	0.6525	0.5516	0.4384

Table S2. Bootstrap 95% confidence intervals for regression coefficients

Response	Coefficient	Estimate	CI_2.5%	CI_97.5%
**CutProfile**	Feed	0.0002	1.4e-05	0.0004
**CutProfile**	Gas	-0.016667	-0.188958	0.133769
**CutProfile**	Intercept	4.533333	4.13565	5.220711
**Depth**	Feed	-0.00445	-0.005983	-0.003731
**Depth**	Gas	-0.016667	-0.744587	0.71225
**Depth**	Intercept	21.964444	18.896618	24.036458
**HAZ**	Feed	-0.000275	-0.0003	-0.00025
**HAZ**	Gas	-0.066667	-0.094335	-0.042306
**HAZ**	Intercept	1.516667	1.43773	1.569652
**SurfaceProfile**	Feed	0.00095	0.000575	0.00144
**SurfaceProfile**	Gas	0.466667	0.119961	0.838916
**SurfaceProfile**	Intercept	2.166667	1.0	3.785354
